# Supplementary material for: Inequalities in antenatal care coverage and quality: an analysis from 63 low and middle-income countries using the ANCq content-qualified coverage indicator
Source: Int J Equity Health. 2021 Apr 17;20:102. doi: 10.1186/s12939-021-01440-3 (PMC8052706; doi:10.1186/s12939-021-01440-3)
Supplement: Supplementary file 1 — Additional file 1. [file 12939_2021_1440_MOESM1_ESM.docx]

## **Supplementary material**

##
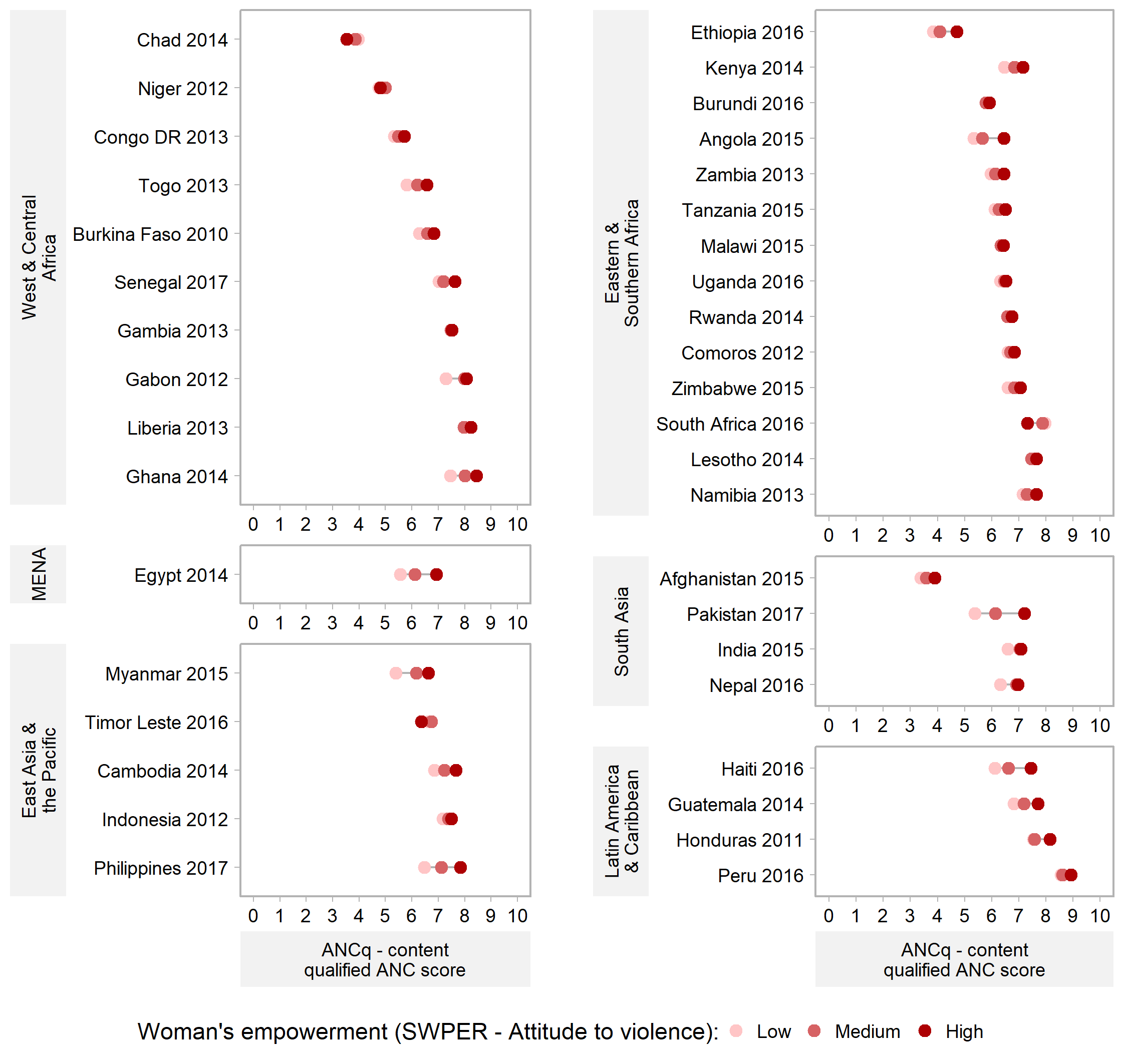


**Figure S1.** Equiplots of ANCq score by SWPER – Attitude to violence domain. Source: DHS, 2010-2017


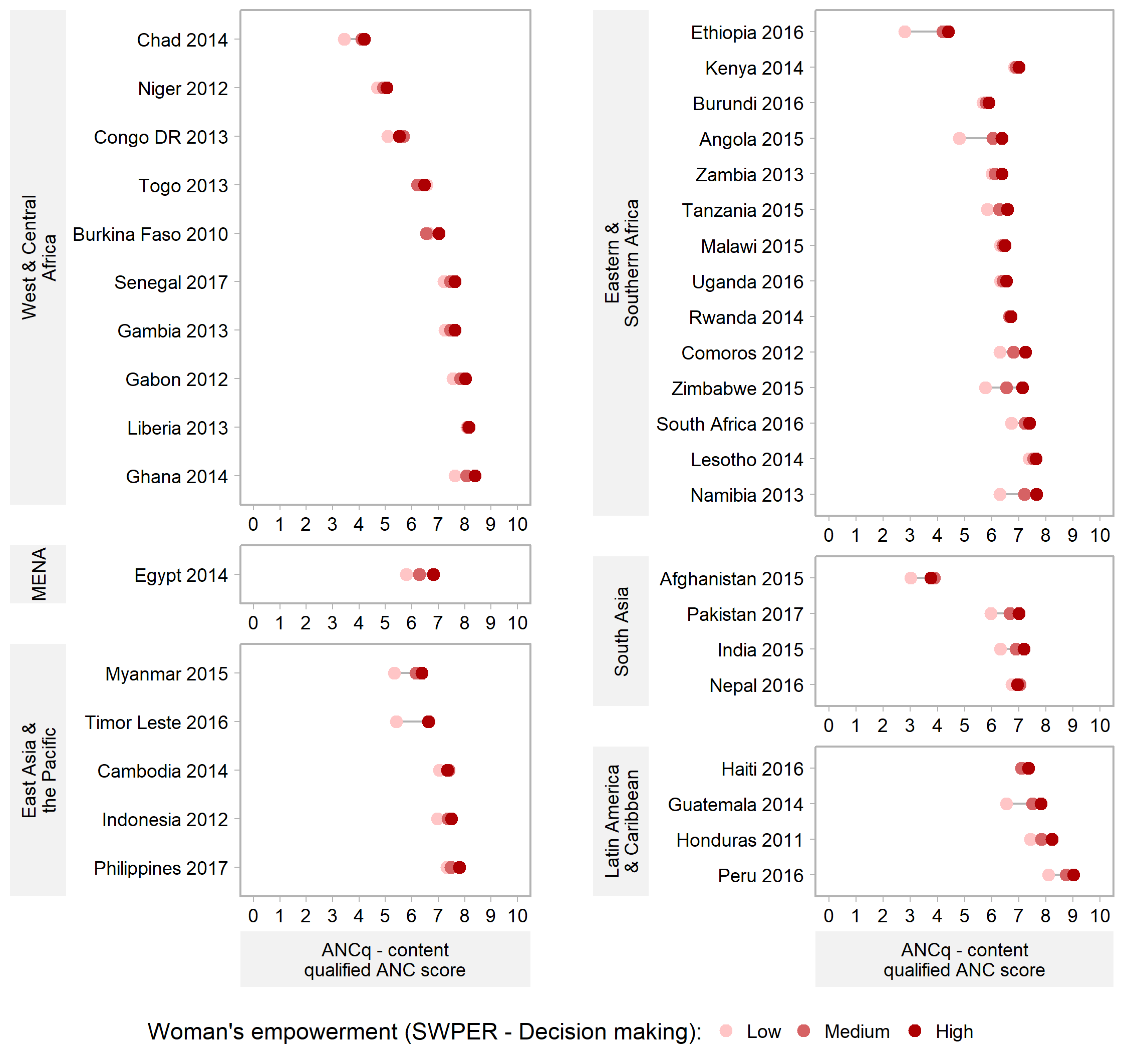


**Figure S2.** Equiplots of ANCq score by SWPER – Decision-making domain. Source: DHS, 2010-2017


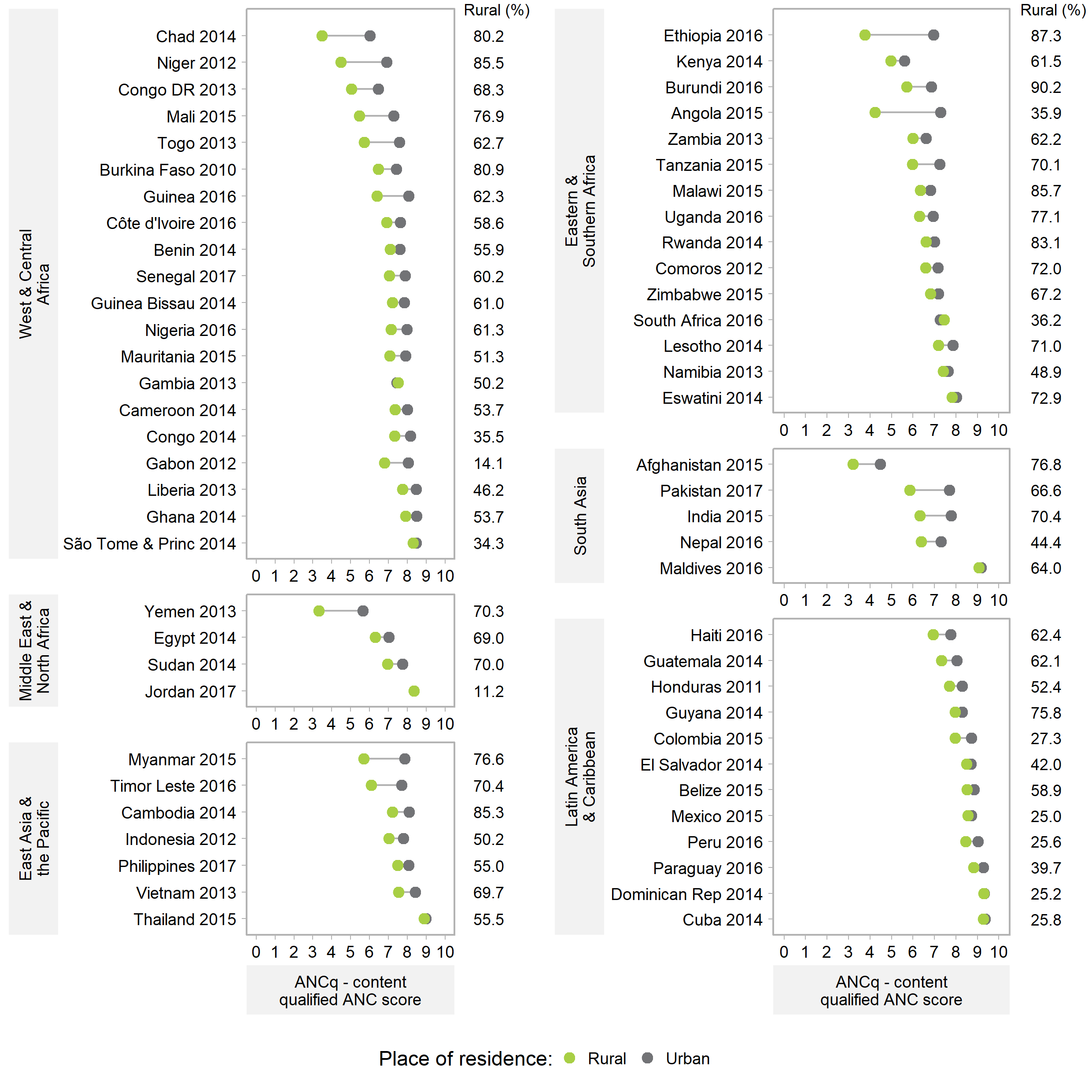


**Figure S3.** Equiplots of ANCq score by place of residence. Source: DHS and MICS, 2010-2017.


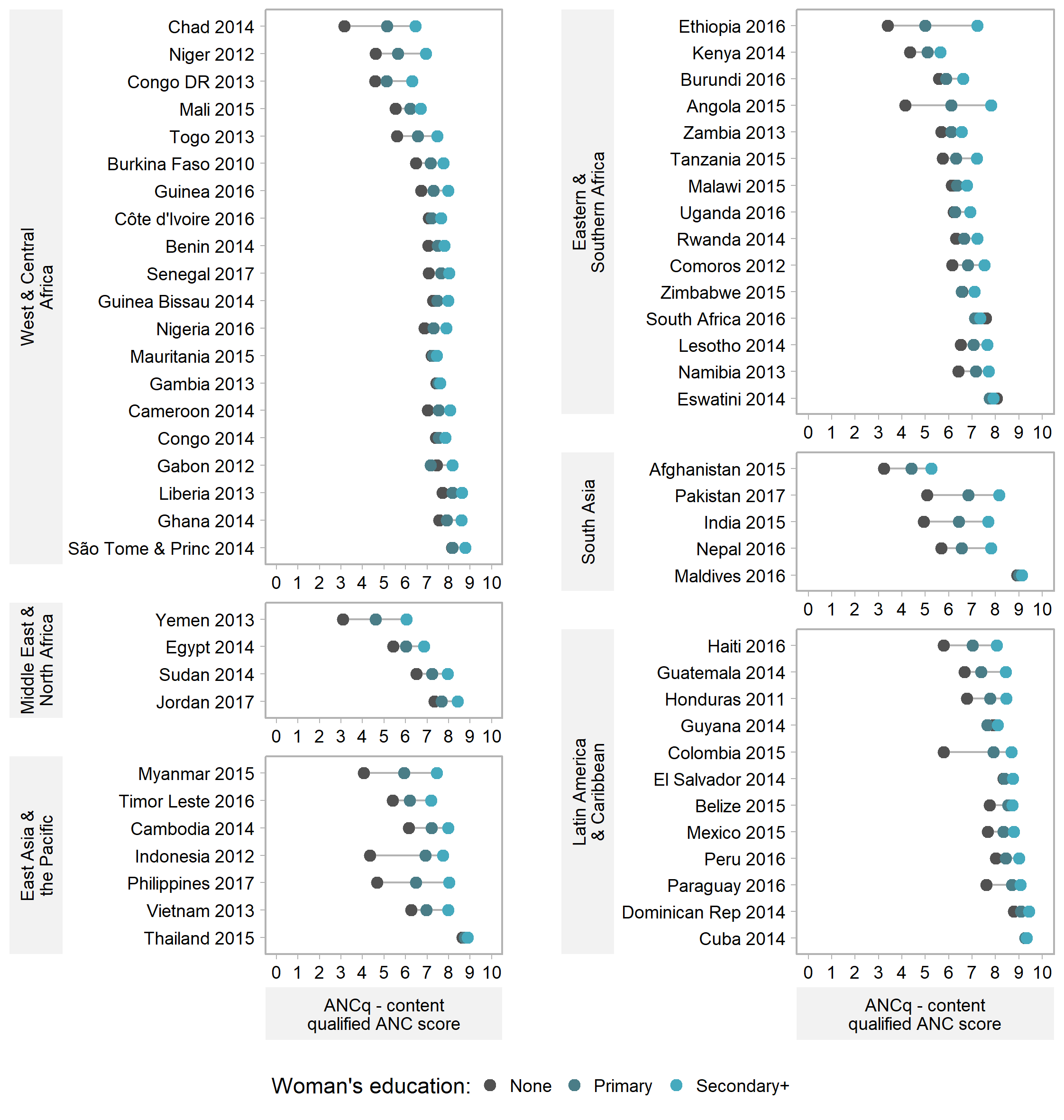


**Figure S4.** Equiplots of ANCq score by woman’s education level. Source: DHS and MICS, 2010-2017.


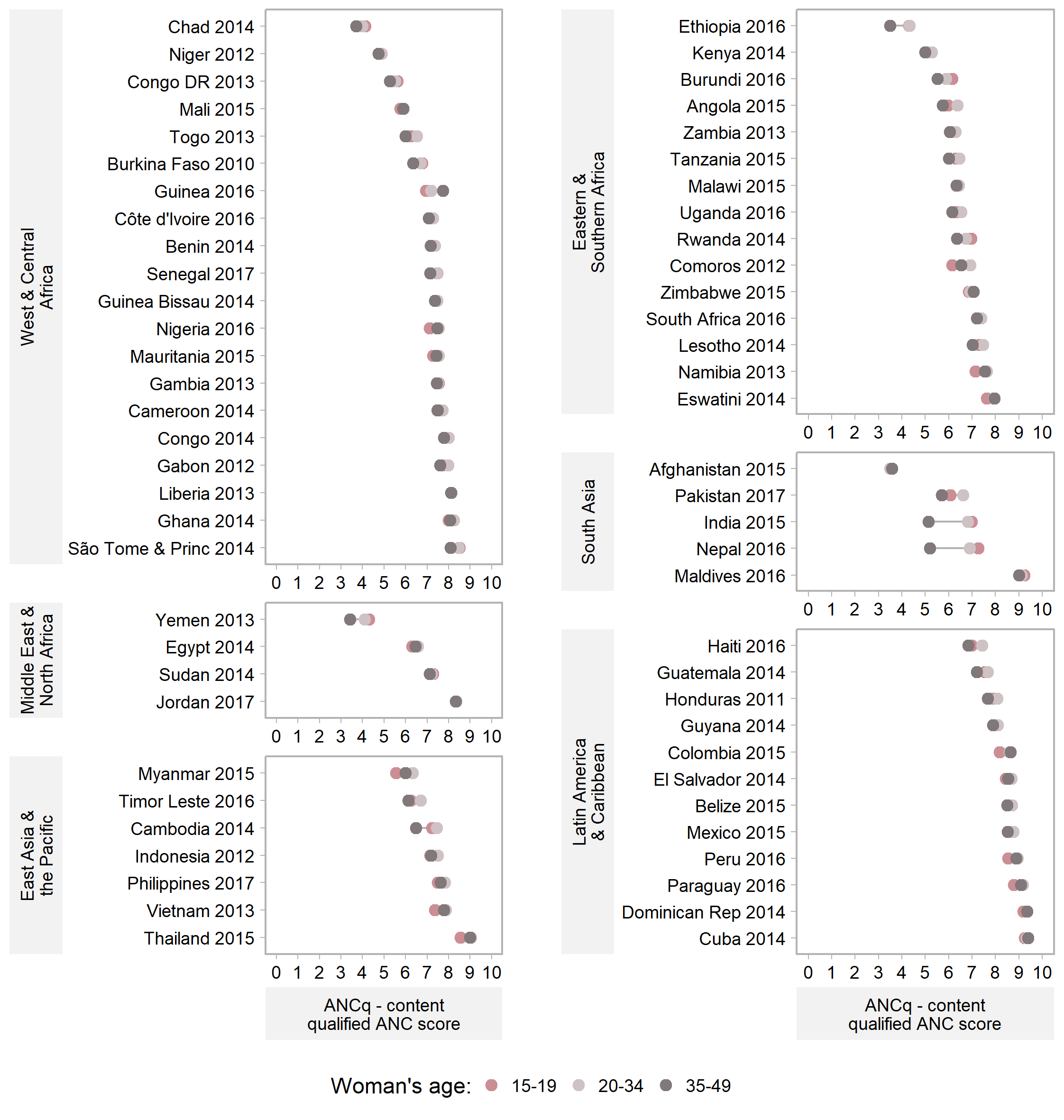


**Figure S5.** Equiplots of ANCq score by woman’s age at childbirth. Source: DHS and MICS, 2010-2017.


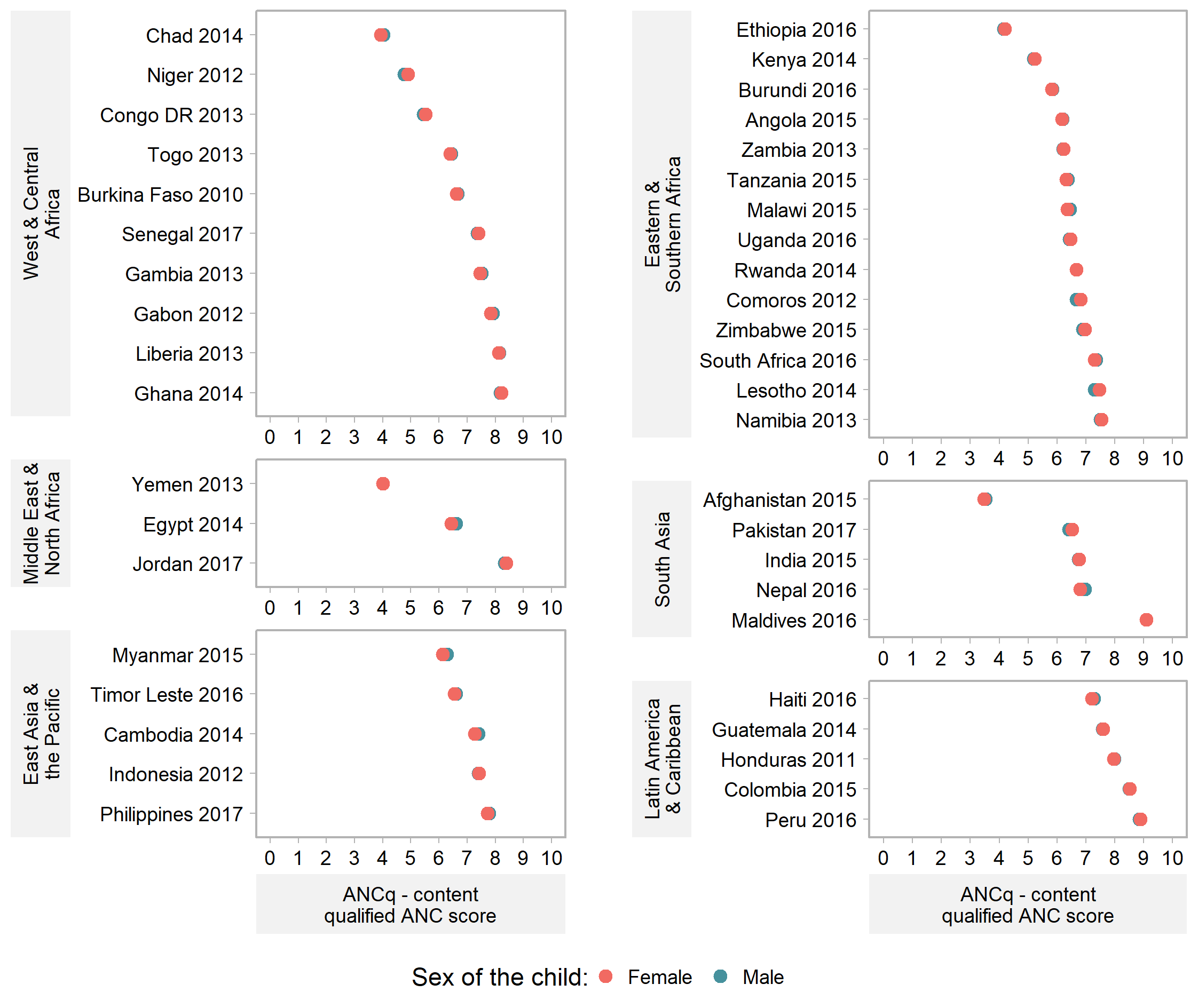


**Figure S6.** Equiplots of ANCq score by sex of the child. Source: DHS, 2010-2017.

**Table S1.** Coverage and Slope Index of Inequality (absolute inequalities) of ANCq components for 12 LMICs. Source: DHS and MICS, 2010-2017.

| **Country** | **Year** | **Wealth quintile** | **ANCq** | **ANCq components** | | | | | | |
| --- | --- | --- | --- | --- | --- | --- | --- | --- | --- | --- |
|  |  |  |  | **ANC visits** | **Skilled** | **First trimester** | **Blood sample** | **Urine sample** | **Blood pressure** | **Tetanus** |
|  |  |  | **Mean (SII)** | **Mean (SII)** | **% (SII)** | **% (SII)** | **% (SII)** | **% (SII)** | **% (SII)** | **% (SII)** |
| **Low and middle ANCq score and high inequality** | | | | | | | | | | |
| Angola | 2015 | Q1 (poorest) | **3.8** | 2.4 | 55.0 | 24.2 | 36.9 | 32.0 | 42.2 | 35.3 |
|  |  | Q5 (wealthiest) | **8.3** | 5.9 | 98.0 | 66.6 | 96.2 | 95.8 | 95.3 | 77.5 |
|  |  | All | **6.2 (6.0)** | 4.1 (4.5) | 81.4 (54.6) | 40.1 (53.1) | 69.8 (81.5) | 67.3 (87.8) | 70.6 (71.1) | 56.1 (54.1) |
| Ethiopia | 2016 | Q1 (poorest) | **2.9** | 1.6 | 47.0 | 14.0 | 28.8 | 25.5 | 32.9 | 30.7 |
|  |  | Q5 (wealthiest) | **6.4** | 3.9 | 85.0 | 37.9 | 77.8 | 73.5 | 76.8 | 55.5 |
|  |  | All | **4.2 (3.7)** | 2.4 (2.5) | 62.3 (41.0) | 20.3 (23.5) | 45.6 (50.1) | 41.5 (50.4) | 47.3 (46.6) | 41.0 (28.6) |
| Afghanistan | 2015 | Q1 (poorest) | **2.9** | 1.4 | 50.0 | 22.2 | 7.9 | 13.4 | 42.8 | 33.1 |
|  |  | Q5 (wealthiest) | **4.8** | 2.9 | 76.0 | 44.7 | 32.5 | 37.4 | 62.5 | 33.8 |
|  |  | All | **3.5 (2.4)** | 1.9 (1.7) | 57.9 (32.2) | 30.1 (27.6) | 17.9 (29.8) | 24.0 (31.0) | 48.1 (25.4) | 33.3 (1.9) |
| **High ANCq score and high inequality** | | | | | | | | | | |
| India | 2015 | Q1 (poorest) | **4.7** | 2.5 | 57.0 | 37.6 | 45.5 | 47.3 | 49.2 | 78.2 |
|  |  | Q5 (wealthiest) | **8.4** | 7.0 | 94.1 | 77.3 | 92.2 | 91.8 | 92.9 | 87.6 |
|  |  | All | **6.8 (4.6)** | 4.8 (5.6) | 79.2 (46.1) | 58.5 (49.1) | 72.8 (58.0) | 73.3 (55.2) | 74.5 (54.3) | 83.0 (10.6) |
| Haiti | 2016 | Q1 (poorest) | **6.0** | 3.5 | 81.6 | 42.5 | 62.3 | 60.8 | 76.7 | 57.9 |
|  |  | Q5 (wealthiest) | **8.5** | 6.7 | 97.4 | 79.1 | 95.9 | 95.5 | 97.0 | 69.4 |
|  |  | All | **7.3 (3.0)** | 4.8 (3.6) | 91.0 (18.6) | 58.5 (44.6) | 80.4 (41.5) | 78.9 (41.5) | 88.2 (24.9) | 64.9 (13.5) |
| Vietnam | 2013 | Q1 (poorest) | **6.5** | 4.0 | 99.0 | 58.4 | 39.4 | 54.2 | 73.9 | 59.7 |
|  |  | Q5 (wealthiest) | **8.9** | 8.3 | 100.0 | 98.4 | 85.7 | 93.0 | 96.2 | 60.3 |
|  |  | All | **7.8 (2.8)** | 6.2 (5.4) | 99.8 (0.9) | 84.4 (45.3) | 64.3 (52.5) | 75.0 (38.6) | 85.7 (26.1) | 61.4 (4.1) |
| **Low and middle ANCq score and low inequality** | | | | | | | | | | |
| Kenya | 2014 | Q1 (poorest) | **4.6** | 3.3 | 89.7 | 13.0 | 38.9 | 32.7 | 37.9 | 20.1 |
|  |  | Q5 (wealthiest) | **5.8** | 4.9 | 99.0 | 31.3 | 45.6 | 45.4 | 45.7 | 29.3 |
|  |  | All | **5.2 (1.5)** | 4.0 (2.0) | 95.9 (10.6) | 19.2 (21.2) | 44.0 (8.5) | 40.7 (16.1) | 43.1 (10.7) | 24.3 (11.9) |
| Burundi | 2016 | Q1 (poorest) | **5.6** | 3.4 | 98.3 | 45.9 | 83.4 | 18.2 | 37.4 | 28.9 |
|  |  | Q5 (wealthiest) | **6.5** | 3.7 | 99.4 | 56.4 | 89.1 | 49.6 | 69.5 | 35.7 |
|  |  | All | **5.8 (0.9)** | 3.5 (0.2) | 99.2 (1.4) | 47.3 (6.9) | 85.6 (4.8) | 26.8 (31.3) | 47.4 (34.4) | 28.5 (5.6) |
| Malawi | 2015 | Q1 (poorest) | **6.2** | 3.6 | 93.1 | 21.5 | 89.0 | 28.9 | 78.0 | 72.6 |
|  |  | Q5 (wealthiest) | **6.8** | 4.0 | 96.7 | 26.6 | 93.3 | 41.4 | 88.7 | 78.9 |
|  |  | All | **6.4 (0.6)** | 3.7 (0.4) | 94.8 (4.1) | 23.9 (5.9) | 90.8 (5.1) | 31.7 (12.6) | 81.7 (12.6) | 73.0 (3.5) |
| **High ANCq score and low inequality** | | | | | | | | | | |
| Dominican Rep | 2014 | Q1 (poorest) | **9.0** | 8.4 | 99.6 | 73.3 | 99.5 | 98.6 | 99.6 | 78.6 |
|  |  | Q5 (wealthiest) | **9.6** | 11.2 | 99.7 | 91.5 | 99.6 | 99.5 | 100.0 | 79.8 |
|  |  | All | **9.3 (0.7)** | 9.7 (3.3) | 99.6 (0.3) | 83.0 (22.2) | 99.7 (0.1) | 99.2 (0.9) | 99.8 (0.6) | 79.4 (2.3) |
| Jordan | 2017 | Q1 (poorest) | **8.1** | 7.8 | 96.4 | 84.2 | 90.8 | 90.0 | 92.2 | 10.5 |
|  |  | Q5 (wealthiest) | **8.5** | 9.8 | 96.5 | 83.6 | 95.2 | 94.9 | 95.9 | 15.6 |
|  |  | All | **8.4 (0.6)** | 8.8 (2.4) | 97.5 (1.3) | 84.9 (0.3) | 94.1 (6.7) | 93.2 (6.9) | 95.0 (5.4) | 10.0 (3.1) |
| Thailand | 2015 | Q1 (poorest) | **8.7** | 8.4 | 100.0 | 69.7 | 99.6 | 99.0 | 100.0 | 40.2 |
|  |  | Q5 (wealthiest) | **9.1** | 8.7 | 100.0 | 94.5 | 99.9 | 99.2 | 99.5 | 56.8 |
|  |  | All | **8.9 (0.4)** | 8.5 (0.0) | 100 (0.0) | 80.0 (29.8) | 99.7 (0.4) | 99.3 (-0.2) | 99.9 (-0.4) | 52.3 (17.8) |

SII: Slope Index of Inequality

**Table S2.** Means and 95% confidence intervals of ANCq score by wealth quintiles. Source: DHS and MICS, 2010-2017.

| **Country** | **Year** | **Source** | **Income Group** | **Wealth quintiles** | | | | | | | | |
| --- | --- | --- | --- | --- | --- | --- | --- | --- | --- | --- | --- | --- |
|  |  |  |  | **Q1 (Poorest)** |  | **Q2** |  | **Q3** |  | **Q4** |  | **Q5 (Wealthiest)** |
|  |  |  |  | **ANCq Mean (95%CI)** |  | **ANCq Mean (95%CI)** |  | **ANCq Mean (95%CI)** |  | **ANCq Mean (95%CI)** |  | **ANCq Mean (95%CI)** |
| **West & Central Africa** | |  |  |  |  |  |  |  |  |  |  |  |
| Chad | 2014 | DHS | Low | 3.4 (3.1-3.7) |  | 3.5 (3.3-3.7) |  | 3.4 (3.2-3.6) |  | 3.5 (3.2-3.8) |  | 6.2 (6.0-6.3) |
| Niger | 2012 | DHS | Low | 3.8 (3.6-4.0) |  | 4.3 (4.1-4.5) |  | 4.6 (4.4-4.7) |  | 4.8 (4.6-5.1) |  | 6.5 (6.4-6.7) |
| Congo DR | 2013 | DHS | Low | 4.4 (4.2-4.7) |  | 5.0 (4.9-5.2) |  | 5.3 (5.1-5.5) |  | 5.8 (5.7-6.0) |  | 6.9 (6.7-7.0) |
| Mali | 2015 | MICS | Low | 5.0 (4.8-5.2) |  | 5.3 (5.1-5.4) |  | 5.3 (5.1-5.5) |  | 5.9 (5.7-6.1) |  | 7.4 (7.2-7.5) |
| Togo | 2013 | DHS | Low | 5.3 (5.1-5.6) |  | 5.4 (5.1-5.6) |  | 5.9 (5.7-6.1) |  | 7.3 (7.1-7.4) |  | 7.9 (7.8-8.0) |
| Burkina Faso | 2010 | DHS | Low | 5.9 (5.7-6.1) |  | 6.3 (6.2-6.4) |  | 6.5 (6.4-6.7) |  | 6.9 (6.8-7.0) |  | 7.4 (7.4-7.5) |
| Guinea | 2016 | MICS | Low | 6.0 (5.8-6.2) |  | 6.3 (6.1-6.4) |  | 6.8 (6.6-7.0) |  | 7.6 (7.4-7.8) |  | 8.3 (8.1-8.4) |
| Nigeria | 2016 | MICS | Lower-middle | 6.4 (6.2-6.6) |  | 6.8 (6.7-6.9) |  | 7.3 (7.2-7.4) |  | 7.7 (7.6-7.8) |  | 8.3 (8.2-8.3) |
| Senegal | 2017 | DHS | Low | 6.5 (6.4-6.7) |  | 7.0 (6.9-7.1) |  | 7.4 (7.3-7.5) |  | 7.8 (7.7-7.9) |  | 8.1 (8.1-8.2) |
| Benin | 2014 | MICS | Low | 6.6 (6.5-6.8) |  | 6.8 (6.7-7.0) |  | 7.2 (7.1-7.4) |  | 7.5 (7.3-7.6) |  | 8.2 (8.1-8.3) |
| Gabon | 2012 | DHS | Upper-middle | 6.7 (6.4-6.9) |  | 7.8 (7.6-8.0) |  | 8.0 (7.8-8.2) |  | 8.4 (8.1-8.6) |  | 8.3 (8.1-8.5) |
| Côte d'Ivoire | 2016 | MICS | Lower-middle | 6.7 (6.5-6.8) |  | 6.9 (6.7-7.0) |  | 7.1 (7.0-7.2) |  | 7.6 (7.5-7.8) |  | 8.0 (7.8-8.1) |
| Mauritania | 2015 | MICS | Lower-middle | 6.7 (6.5-6.9) |  | 7.0 (6.8-7.2) |  | 7.4 (7.2-7.5) |  | 7.7 (7.6-7.8) |  | 8.2 (8.1-8.3) |
| Cameroon | 2014 | MICS | Lower-middle | 7.0 (6.7-7.2) |  | 7.2 (7.1-7.4) |  | 7.6 (7.5-7.7) |  | 7.8 (7.8-7.9) |  | 8.3 (8.2-8.4) |
| Congo | 2014 | MICS | Lower-middle | 7.1 (7.0-7.2) |  | 7.7 (7.6-7.8) |  | 8.0 (7.9-8.1) |  | 8.2 (8.1-8.3) |  | 8.3 (8.2-8.5) |
| Guinea Bissau | 2014 | MICS | Low | 7.1 (7.0-7.2) |  | 7.1 (7.0-7.3) |  | 7.2 (7.1-7.4) |  | 7.7 (7.6-7.8) |  | 8.1 (7.9-8.3) |
| Ghana | 2014 | DHS | Lower-middle | 7.3 (7.0-7.7) |  | 7.8 (7.6-7.9) |  | 8.2 (8.1-8.4) |  | 8.5 (8.4-8.6) |  | 8.9 (8.8-9.0) |
| Liberia | 2013 | DHS | Low | 7.4 (7.2-7.6) |  | 7.7 (7.6-7.9) |  | 8.2 (8.0-8.3) |  | 8.6 (8.4-8.7) |  | 8.8 (8.6-9.0) |
| Gambia | 2013 | DHS | Low | 7.4 (7.3-7.5) |  | 7.5 (7.4-7.6) |  | 7.3 (7.2-7.5) |  | 7.4 (7.3-7.5) |  | 7.6 (7.5-7.8) |
| São Tome & Principe | 2014 | MICS | Lower-middle | 8.0 (7.7-8.2) |  | 8.3 (8.1-8.4) |  | 8.4 (8.2-8.6) |  | 8.5 (8.2-8.7) |  | 8.8 (8.5-9.0) |
| **Eastern & Southern Africa** | |  |  |  |  |  |  |  |  |  |  |  |
| Ethiopia | 2016 | DHS | Low | 2.9 (2.6-3.2) |  | 3.5 (3.2-3.9) |  | 3.9 (3.6-4.2) |  | 4.5 (4.1-4.8) |  | 6.4 (6.1-6.7) |
| Angola | 2015 | DHS | Upper-middle | 3.7 (3.4-4.0) |  | 4.7 (4.5-4.9) |  | 6.7 (6.5-6.9) |  | 7.7 (7.6-7.9) |  | 8.3 (8.1-8.4) |
| Kenya | 2014 | DHS | Lower-middle | 4.5 (4.4-4.6) |  | 4.9 (4.8-5.0) |  | 5.1 (5.1-5.2) |  | 5.4 (5.3-5.5) |  | 5.7 (5.6-5.9) |
| Burundi | 2016 | DHS | Low | 5.5 (5.4-5.6) |  | 5.7 (5.6-5.7) |  | 5.7 (5.6-5.8) |  | 5.7 (5.6-5.8) |  | 6.5 (6.3-6.6) |
| Tanzania | 2015 | DHS | Low | 5.7 (5.5-5.8) |  | 5.8 (5.6-5.9) |  | 6.1 (6.0-6.2) |  | 6.6 (6.5-6.8) |  | 7.5 (7.3-7.6) |
| Zambia | 2013 | DHS | Lower-middle | 5.7 (5.6-5.8) |  | 5.9 (5.9-6.0) |  | 6.2 (6.1-6.2) |  | 6.4 (6.3-6.5) |  | 6.9 (6.8-7.0) |
| Comoros | 2012 | DHS | Low | 5.8 (5.4-6.2) |  | 6.3 (6.0-6.7) |  | 7.1 (6.8-7.4) |  | 7.1 (6.9-7.4) |  | 7.5 (7.2-7.8) |
| Uganda | 2016 | DHS | Low | 6.1 (6-6.2.0) |  | 6.1 (6.0-6.2) |  | 6.3 (6.2-6.4) |  | 6.5 (6.4-6.6) |  | 7.0 (6.9-7.1) |
| Malawi | 2015 | DHS | Low | 6.2 (6.1-6.3) |  | 6.3 (6.2-6.4) |  | 6.3 (6.2-6.3) |  | 6.4 (6.3-6.5) |  | 6.8 (6.7-6.9) |
| Zimbabwe | 2015 | DHS | Low | 6.5 (6.2-6.7) |  | 6.7 (6.5-7.0) |  | 6.9 (6.7-7.2) |  | 6.8 (6.7-7.0) |  | 7.6 (7.4-7.7) |
| Rwanda | 2014 | DHS | Low | 6.5 (6.4-6.6) |  | 6.5 (6.4-6.6) |  | 6.6 (6.5-6.7) |  | 6.6 (6.5-6.7) |  | 7.0 (6.9-7.1) |
| Lesotho | 2014 | DHS | Lower-middle | 6.8 (6.6-7.1) |  | 6.9 (6.7-7.2) |  | 7.2 (7.0-7.5) |  | 7.6 (7.4-7.8) |  | 8.1 (7.8-8.3) |
| South Africa | 2016 | DHS | Upper-middle | 7.1 (6.8-7.4) |  | 7.2 (6.8-7.5) |  | 7.3 (7.1-7.6) |  | 7.5 (7.3-7.7) |  | 7.4 (7.1-7.8) |
| Namibia | 2013 | DHS | Upper-middle | 7.2 (7.0-7.4) |  | 7.3 (7.1-7.5) |  | 7.5 (7.3-7.7) |  | 7.6 (7.5-7.8) |  | 7.8 (7.6-8.0) |
| Eswatini | 2014 | MICS | Lower-middle | 7.7 (7.6-7.8) |  | 7.7 (7.5-7.8) |  | 7.7 (7.5-7.9) |  | 7.9 (7.7-8.2) |  | 8.2 (7.9-8.4) |
| **Middle East & North Africa** | |  |  |  |  |  |  |  |  |  |  |  |
| Yemen | 2013 | DHS | Lower-middle | 2.4 (2.2-2.7) |  | 2.8 (2.6-3.0) |  | 3.6 (3.4-3.8) |  | 4.8 (4.6-5.0) |  | 6.2 (6.0-6.4) |
| Egypt | 2014 | DHS | Lower-middle | 5.5 (5.3-5.7) |  | 6.0 (5.9-6.1) |  | 6.5 (6.4-6.6) |  | 7.0 (6.8-7.1) |  | 7.4 (7.3-7.6) |
| Sudan | 2014 | MICS | Lower-middle | 6.2 (6.0-6.4) |  | 6.7 (6.5-6.8) |  | 7.2 (7.0-7.3) |  | 7.5 (7.4-7.7) |  | 8.1 (8.0-8.2) |
| Jordan | 2017 | DHS | Upper-middle | 8.0 (7.9-8.1) |  | 8.3 (8.1-8.4) |  | 8.5 (8.4-8.6) |  | 8.5 (8.3-8.6) |  | 8.5 (8.2-8.8) |
| **South Asia** |  |  |  |  |  |  |  |  |  |  |  |  |
| Afghanistan | 2015 | DHS | Low | 2.8 (2.6-3.0) |  | 2.8 (2.6-3.1) |  | 3.2 (2.7-3.6) |  | 3.8 (3.6-4.1) |  | 4.7 (4.5-5.0) |
| Pakistan | 2017 | DHS | Lower-middle | 4.2 (3.9-4.5) |  | 5.3 (5.1-5.6) |  | 6.6 (6.4-6.9) |  | 7.7 (7.5-7.9) |  | 8.5 (8.4-8.6) |
| India | 2015 | DHS | Lower-middle | 4.6 (4.6-4.7) |  | 6.3 (6.2-6.3) |  | 7.2 (7.2-7.3) |  | 7.8 (7.7-7.8) |  | 8.3 (8.3-8.4) |
| Nepal | 2016 | DHS | Low | 5.7 (5.4-5.9) |  | 6.5 (6.2-6.7) |  | 6.8 (6.6-7.1) |  | 7.4 (7.2-7.6) |  | 8.2 (8.0-8.4) |
| Maldives | 2016 | DHS | Upper-middle | 9.0 (8.9-9.1) |  | 9.0 (8.8-9.1) |  | 9.1 (9.0-9.2) |  | 8.9 (8.7-9.1) |  | 9.3 (9.2-9.5) |
| **East Asia & the Pacific** | |  |  |  |  |  |  |  |  |  |  |  |
| Myanmar | 2015 | DHS | Lower-middle | 4.7 (4.4-5.1) |  | 5.7 (5.4-6.0) |  | 6.3 (5.9-6.6) |  | 7.1 (6.8-7.3) |  | 8.2 (7.9-8.4) |
| Timor Leste | 2016 | DHS | Lower-middle | 5.4 (5.1-5.7) |  | 5.9 (5.6-6.1) |  | 6.2 (6.0-6.5) |  | 7.2 (7.0-7.4) |  | 7.9 (7.7-8.1) |
| Indonesia | 2012 | DHS | Lower-middle | 6.2 (6.0-6.3) |  | 7.2 (7.1-7.4) |  | 7.5 (7.4-7.6) |  | 7.9 (7.8-7.9) |  | 8.0 (8.0-8.1) |
| Vietnam | 2013 | MICS | Lower-middle | 6.5 (6.2-6.7) |  | 7.4 (7.1-7.6) |  | 7.6 (7.4-7.9) |  | 8.3 (8.1-8.5) |  | 8.8 (8.7-9.0) |
| Cambodia | 2014 | DHS | Low | 6.5 (6.2-6.8) |  | 7.0 (6.8-7.2) |  | 7.3 (7.1-7.5) |  | 7.7 (7.6-7.8) |  | 8.1 (8.0-8.2) |
| Philippines | 2017 | DHS | Lower-middle | 6.5 (6.3-6.8) |  | 7.4 (7.3-7.6) |  | 7.9 (7.8-8.1) |  | 8.5 (8.3-8.6) |  | 8.9 (8.7-9.0) |
| Thailand | 2015 | MICS | Upper-middle | 8.7 (8.5-8.9) |  | 8.8 (8.6-9.0) |  | 8.9 (8.8-9.1) |  | 9.0 (8.8-9.1) |  | 9.1 (8.9-9.2) |
| **Latin America & Caribbean** | |  |  |  |  |  |  |  |  |  |  |  |
| Haiti | 2016 | DHS | Low | 5.9 (5.6-6.3) |  | 6.8 (6.5-7.0) |  | 7.4 (7.2-7.7) |  | 7.8 (7.6-7.9) |  | 8.5 (8.3-8.7) |
| Guatemala | 2014 | DHS | Lower-middle | 6.7 (6.5-6.9) |  | 7.2 (7.0-7.3) |  | 7.6 (7.4-7.7) |  | 8.2 (8.1-8.3) |  | 8.6 (8.5-8.7) |
| Honduras | 2011 | DHS | Lower-middle | 7.2 (7.1-7.3) |  | 7.6 (7.5-7.7) |  | 8.0 (7.9-8.2) |  | 8.3 (8.2-8.4) |  | 8.7 (8.6-8.8) |
| Guyana | 2014 | MICS | Lower-middle | 7.3 (7.1-7.5) |  | 8.1 (8.0-8.2) |  | 8.3 (8.1-8.5) |  | 8.3 (8.1-8.4) |  | 8.6 (8.4-8.8) |
| Colombia | 2015 | DHS | Upper-middle | 7.8 (7.7-8.0) |  | 8.4 (8.3-8.5) |  | 8.6 (8.5-8.7) |  | 8.8 (8.7-8.9) |  | 9.1 (8.9-9.3) |
| Belize | 2015 | MICS | Upper-middle | 8.2 (8.0-8.3) |  | 8.6 (8.3-8.8) |  | 8.7 (8.4-8.9) |  | 8.8 (8.7-9.0) |  | 9.0 (8.8-9.2) |
| Peru | 2016 | DHS | Upper-middle | 8.2 (8.1-8.4) |  | 8.8 (8.8-8.9) |  | 9.0 (8.9-9.0) |  | 9.1 (9.0-9.1) |  | 9.1 (9.1-9.2) |
| El Salvador | 2014 | MICS | Lower-middle | 8.3 (8.2-8.4) |  | 8.4 (8.3-8.5) |  | 8.7 (8.5-8.8) |  | 8.7 (8.6-8.8) |  | 8.9 (8.8-9.0) |
| Mexico | 2015 | MICS | Upper-middle | 8.4 (8.2-8.5) |  | 8.6 (8.4-8.7) |  | 8.7 (8.5-8.9) |  | 8.8 (8.7-9.0) |  | 9.0 (8.7-9.2) |
| Paraguay | 2016 | MICS | Upper-middle | 8.6 (8.4-8.7) |  | 8.9 (8.8-9.1) |  | 9.2 (9.1-9.3) |  | 9.3 (9.2-9.5) |  | 9.5 (9.4-9.6) |
| Dominican Rep | 2014 | MICS | Upper-middle | 9.0 (8.9-9.1) |  | 9.2 (9.1-9.3) |  | 9.4 (9.3-9.4) |  | 9.4 (9.4-9.5) |  | 9.5 (9.4-9.6) |

**Table S3.** Means and 95% confidence intervals of ANCq score by SWPER - Social independence domain. Source: DHS, 2010-2017.

| **Country** | **Year** | **Income Group** | **Woman's Empowerment (SWPER - Social Independence)** | | | | |
| --- | --- | --- | --- | --- | --- | --- | --- |
|  |  |  | **Low** |  | **Medium** |  | **High** |
|  |  |  | **ANCq Mean (95%CI)** |  | **ANCq Mean (95%CI)** |  | **ANCq Mean (95%CI)** |
| **West & Central Africa** | |  |  |  |  |  |  |
| Chad | 2014 | Low | 3.6 (3.5-3.7) |  | 4.2 (4.0-4.4) |  | 5.1 (4.7-5.4) |
| Niger | 2012 | Low | 4.6 (4.6-4.7) |  | 4.9 (4.7-5.0) |  | 6.2 (5.9-6.5) |
| Congo DR | 2013 | Low | 5.0 (4.9-5.1) |  | 5.5 (5.4-5.6) |  | 6.2 (6.0-6.3) |
| Togo | 2013 | Low | 5.7 (5.5-5.8) |  | 6.4 (6.3-6.5) |  | 7.3 (7.2-7.4) |
| Burkina Faso | 2010 | Low | 6.4 (6.4-6.5) |  | 6.8 (6.7-6.8) |  | 7.4 (7.2-7.5) |
| Senegal | 2017 | Low | 7.0 (6.9-7.1) |  | 7.2 (7.2-7.3) |  | 7.9 (7.8-7.9) |
| Gabon | 2012 | Upper-middle | 7.1 (6.8-7.4) |  | 7.9 (7.7-8.1) |  | 8.1 (7.9-8.3) |
| Gambia | 2013 | Low | 7.4 (7.3-7.4) |  | 7.5 (7.4-7.5) |  | 7.6 (7.5-7.8) |
| Liberia | 2013 | Low | 7.9 (7.8-8.0) |  | 8.2 (8.1-8.3) |  | 8.5 (8.4-8.7) |
| Ghana | 2014 | Lower-middle | 7.8 (7.6-7.9) |  | 8.0 (7.9-8.2) |  | 8.6 (8.5-8.7) |
| **Eastern & Southern Africa** | |  |  |  |  |  |  |
| Ethiopia | 2016 | Low | 3.7 (3.6-3.9) |  | 4.3 (4.0-4.5) |  | 5.7 (5.4-6.1) |
| Kenya | 2014 | Lower-middle | 6.3 (6.1-6.4) |  | 6.8 (6.7-6.9) |  | 7.4 (7.3-7.5) |
| Angola | 2015 | Upper-middle | 5.3 (5.2-5.5) |  | 6.2 (6.1-6.4) |  | 7.4 (7.2-7.6) |
| Burundi | 2016 | Low | 5.6 (5.5-5.6) |  | 5.8 (5.7-5.8) |  | 6.1 (6.0-6.2) |
| Zambia | 2013 | Lower-middle | 6.0 (5.9-6.0) |  | 6.2 (6.1-6.2) |  | 6.6 (6.5-6.6) |
| Tanzania | 2015 | Low | 5.8 (5.7-6.0) |  | 6.1 (6.0-6.2) |  | 6.8 (6.7-6.9) |
| Uganda | 2016 | Low | 6.2 (6.1-6.3) |  | 6.3 (6.3-6.4) |  | 6.9 (6.8-6.9) |
| Malawi | 2015 | Low | 6.2 (6.2-6.3) |  | 6.4 (6.3-6.4) |  | 6.7 (6.6-6.8) |
| Rwanda | 2014 | Low | 6.4 (6.2-6.5) |  | 6.5 (6.5-6.6) |  | 6.8 (6.8-6.9) |
| Comoros | 2012 | Low | 6.4 (6.1-6.6) |  | 6.6 (6.3-6.9) |  | 7.2 (7.0-7.4) |
| Zimbabwe | 2015 | Low | 6.1 (5.9-6.4) |  | 6.8 (6.7-6.9) |  | 7.4 (7.3-7.5) |
| Lesotho | 2014 | Lower-middle | 6.8 (6.3-7.3) |  | 7.5 (7.3-7.6) |  | 7.8 (7.6-7.9) |
| South Africa | 2016 | Upper-middle | 6.4 (5.1-7.7) |  | 7.2 (6.8-7.6) |  | 7.4 (7.2-7.6) |
| Namibia | 2013 | Upper-middle | 6.6 (6.3-7.0) |  | 7.1 (6.9-7.4) |  | 7.7 (7.6-7.9) |
| **Middle East & North Africa** | |  |  |  |  |  |  |
| Egypt | 2014 | Lower-middle | 5.4 (5.2-5.6) |  | 6.3 (6.2-6.4) |  | 6.9 (6.9-7.0) |
| **South Asia** |  |  |  |  |  |  |  |
| Afghanistan | 2015 | Low | 3.3 (3.2-3.4) |  | 3.4 (3.3-3.5) |  | 4.4 (4.1-4.6) |
| Pakistan | 2017 | Lower-middle | 5.2 (5.0-5.4) |  | 6.2 (6.1-6.4) |  | 7.7 (7.5-7.8) |
| India | 2015 | Lower-middle | 5.5 (5.4-5.6) |  | 6.6 (6.5-6.7) |  | 7.8 (7.8-7.9) |
| Nepal | 2016 | Low | 5.9 (5.8-6.1) |  | 6.9 (6.8-7.1) |  | 8.0 (7.8-8.1) |
| **East Asia & the Pacific** | |  |  |  |  |  |  |
| Myanmar | 2015 | Lower-middle | 4.7 (4.4-5.0) |  | 5.5 (5.3-5.7) |  | 7.1 (7.0-7.2) |
| Timor Leste | 2016 | Lower-middle | 5.7 (5.5-6.0) |  | 6.3 (6.1-6.4) |  | 7.0 (6.9-7.1) |
| Indonesia | 2012 | Lower-middle | 6.4 (6.2-6.5) |  | 7.2 (7.1-7.3) |  | 7.7 (7.6-7.7) |
| Cambodia | 2014 | Low | 6.4 (6.2-6.6) |  | 7.2 (7.1-7.3) |  | 7.7 (7.6-7.8) |
| Philippines | 2017 | Lower-middle | 6.3 (6.0-6.7) |  | 7.3 (7.2-7.4) |  | 8.0 (8.0-8.1) |
| **Latin America & Caribbean** | |  |  |  |  |  |  |
| Haiti | 2016 | Low | 6.4 (6.2-6.7) |  | 6.9 (6.8-7.1) |  | 7.9 (7.8-8.0) |
| Guatemala | 2014 | Lower-middle | 6.9 (6.8-7.0) |  | 7.5 (7.4-7.6) |  | 8.2 (8.1-8.2) |
| Honduras | 2011 | Lower-middle | 7.5 (7.4-7.6) |  | 8.0 (7.9-8.1) |  | 8.5 (8.4-8.6) |
| Peru | 2016 | Upper-middle | 8.3 (8.1-8.4) |  | 8.7 (8.6-8.7) |  | 9.0 (9.0-9.1) |

**Table S4.** Means and 95% confidence intervals of ANCq score by SWPER - Attitude to violence domain. Source: DHS, 2010-2017.

| **Country** | **Year** | **Income Group** | **Woman's Empowerment (SWPER - Attitude to Violence)** | | | | |
| --- | --- | --- | --- | --- | --- | --- | --- |
|  |  |  | **Low** |  | **Medium** |  | **High** |
|  |  |  | **ANCq Mean (95%CI)** |  | **ANCq Mean (95%CI)** |  | **ANCq Mean (95%CI)** |
| **West & Central Africa** | |  |  |  |  |  |  |
| Chad | 2014 | Low | 3.9 (3.8-4.0) |  | 3.8 (3.6-4.0) |  | 3.5 (3.3-3.6) |
| Niger | 2012 | Low | 4.7 (4.6-4.8) |  | 4.9 (4.8-5.1) |  | 4.8 (4.7-4.9) |
| Congo DR | 2013 | Low | 5.3 (5.2-5.4) |  | 5.4 (5.3-5.5) |  | 5.7 (5.6-5.8) |
| Togo | 2013 | Low | 5.8 (5.6-5.9) |  | 6.2 (6.0-6.4) |  | 6.5 (6.4-6.6) |
| Burkina Faso | 2010 | Low | 6.2 (6.1-6.3) |  | 6.5 (6.5-6.6) |  | 6.8 (6.7-6.8) |
| Senegal | 2017 | Low | 7.0 (6.9-7.0) |  | 7.1 (7.0-7.3) |  | 7.6 (7.5-7.6) |
| Gabon | 2012 | Upper-middle | 7.2 (6.9-7.5) |  | 7.9 (7.7-8.2) |  | 8.0 (7.8-8.2) |
| Gambia | 2013 | Low | 7.4 (7.3-7.5) |  | 7.4 (7.3-7.5) |  | 7.5 (7.4-7.5) |
| Liberia | 2013 | Low | 8.0 (7.9-8.1) |  | 7.9 (7.8-8.1) |  | 8.2 (8.1-8.3) |
| Ghana | 2014 | Lower-middle | 7.4 (7.2-7.6) |  | 8.0 (7.8-8.1) |  | 8.4 (8.3-8.5) |
| **Eastern & Southern Africa** | |  |  |  |  |  |  |
| Ethiopia | 2016 | Low | 3.8 (3.6-4.0) |  | 4.0 (3.8-4.3) |  | 4.7 (4.5-4.9) |
| Kenya | 2014 | Lower-middle | 6.4 (6.3-6.5) |  | 6.8 (6.7-6.9) |  | 7.1 (7.0-7.2) |
| Angola | 2015 | Upper-middle | 5.3 (5.0-5.6) |  | 5.6 (5.3-5.9) |  | 6.4 (6.3-6.5) |
| Burundi | 2016 | Low | 5.7 (5.7-5.8) |  | 5.7 (5.7-5.8) |  | 5.9 (5.8-5.9) |
| Zambia | 2013 | Lower-middle | 5.9 (5.9-6.0) |  | 6.1 (6.0-6.2) |  | 6.4 (6.3-6.5) |
| Tanzania | 2015 | Low | 6.1 (6.0-6.2) |  | 6.2 (6.1-6.4) |  | 6.5 (6.4-6.5) |
| Uganda | 2016 | Low | 6.3 (6.2-6.4) |  | 6.4 (6.3-6.5) |  | 6.5 (6.4-6.5) |
| Malawi | 2015 | Low | 6.4 (6.2-6.5) |  | 6.3 (6.2-6.4) |  | 6.4 (6.4-6.4) |
| Rwanda | 2014 | Low | 6.6 (6.5-6.7) |  | 6.5 (6.4-6.6) |  | 6.7 (6.6-6.8) |
| Comoros | 2012 | Low | 6.5 (6.2-6.9) |  | 6.6 (6.3-7.0) |  | 6.8 (6.6-7.0) |
| Zimbabwe | 2015 | Low | 6.6 (6.3-6.8) |  | 6.8 (6.6-7.0) |  | 7.0 (6.9-7.1) |
| Lesotho | 2014 | Lower-middle | 7.5 (7.3-7.6) |  | 7.4 (7.2-7.6) |  | 7.6 (7.5-7.7) |
| South Africa | 2016 | Upper-middle | 7.9 (7.5-8.3) |  | 7.8 (7.5-8.2) |  | 7.3 (7.1-7.5) |
| Namibia | 2013 | Upper-middle | 7.1 (6.8-7.4) |  | 7.2 (7.0-7.5) |  | 7.6 (7.5-7.7) |
| **Middle East & North Africa** | |  |  |  |  |  |  |
| Egypt | 2014 | Lower-middle | 5.5 (5.4-5.7) |  | 6.1 (5.9-6.2) |  | 6.9 (6.8-6.9) |
| **South Asia** |  |  |  |  |  |  |  |
| Afghanistan | 2015 | Low | 3.3 (3.2-3.4) |  | 3.5 (3.4-3.7) |  | 3.8 (3.6-4.1) |
| Pakistan | 2017 | Lower-middle | 5.3 (5.2-5.5) |  | 6.1 (5.8-6.3) |  | 7.2 (7.0-7.3) |
| India | 2015 | Lower-middle | 6.6 (6.5-6.7) |  | 7.0 (6.9-7.1) |  | 7.0 (7.0-7.1) |
| Nepal | 2016 | Low | 6.3 (5.9-6.7) |  | 6.9 (6.7-7.1) |  | 6.9 (6.8-7.0) |
| **East Asia & the Pacific** | |  |  |  |  |  |  |
| Myanmar | 2015 | Lower-middle | 5.3 (5.1-5.6) |  | 6.1 (5.9-6.3) |  | 6.6 (6.4-6.7) |
| Timor Leste | 2016 | Lower-middle | 6.6 (6.5-6.7) |  | 6.7 (6.5-6.9) |  | 6.3 (6.1-6.6) |
| Indonesia | 2012 | Lower-middle | 7.1 (7.0-7.3) |  | 7.3 (7.2-7.4) |  | 7.5 (7.4-7.5) |
| Cambodia | 2014 | Low | 6.8 (6.6-6.9) |  | 7.2 (7.0-7.3) |  | 7.6 (7.5-7.7) |
| Philippines | 2017 | Lower-middle | 6.4 (5.9-7.0) |  | 7.1 (6.8-7.3) |  | 7.8 (7.7-7.9) |
| **Latin America & Caribbean** | |  |  |  |  |  |  |
| Haiti | 2016 | Low | 6.1 (5.6-6.6) |  | 6.6 (6.3-6.9) |  | 7.4 (7.3-7.5) |
| Guatemala | 2014 | Lower-middle | 6.8 (6.5-7.1) |  | 7.1 (6.9-7.3) |  | 7.7 (7.6-7.7) |
| Honduras | 2011 | Lower-middle | 7.5 (7.3-7.7) |  | 7.5 (7.4-7.7) |  | 8.1 (8.1-8.2) |
| Peru | 2016 | Upper-middle | 8.5 (8.0-9.0) |  | 8.6 (8.3-8.9) |  | 8.9 (8.8-8.9) |

**Table S5.** Means and 95% confidence intervals of ANCq score by SWPER - Decision-making domain. Source: DHS, 2010-2017.

| **Country** | **Year** | **Income Group** | **Woman's Empowerment (SWPER – Decision Making)** | | | | |
| --- | --- | --- | --- | --- | --- | --- | --- |
|  |  |  | **Low** |  | **Medium** |  | **High** |
|  |  |  | **ANCq Mean (95%CI)** |  | **ANCq Mean (95%CI)** |  | **ANCq Mean (95%CI)** |
| **West & Central Africa** | |  |  |  |  |  |  |
| Chad | 2014 | Low | 3.4 (3.3-3.5) |  | 4.1 (3.9-4.2) |  | 4.1 (3.9-4.3) |
| Niger | 2012 | Low | 4.6 (4.5-4.7) |  | 4.9 (4.8-5.0) |  | 5.0 (4.8-5.2) |
| Congo DR | 2013 | Low | 5.0 (4.9-5.2) |  | 5.6 (5.5-5.7) |  | 5.5 (5.4-5.6) |
| Togo | 2013 | Low | 6.5 (6.4-6.7) |  | 6.2 (6.0-6.3) |  | 6.4 (6.3-6.6) |
| Burkina Faso | 2010 | Low | 6.6 (6.5-6.7) |  | 6.5 (6.4-6.6) |  | 7.0 (6.9-7.1) |
| Senegal | 2017 | Low | 7.2 (7.1-7.2) |  | 7.4 (7.3-7.5) |  | 7.6 (7.5-7.7) |
| Gabon | 2012 | Upper-middle | 7.5 (7.2-7.9) |  | 7.8 (7.6-8.0) |  | 8.0 (7.8-8.2) |
| Gambia | 2013 | Low | 7.2 (7.0-7.4) |  | 7.4 (7.3-7.5) |  | 7.6 (7.5-7.6) |
| Liberia | 2013 | Low | 8.0 (7.8-8.3) |  | 8.0 (7.8-8.3) |  | 8.1 (8.0-8.2) |
| Ghana | 2014 | Lower-middle | 7.6 (7.3-7.9) |  | 8.0 (7.9-8.1) |  | 8.3 (8.3-8.4) |
| **Eastern & Southern Africa** | |  |  |  |  |  |  |
| Ethiopia | 2016 | Low | 2.7 (2.4-3.1) |  | 4.1 (3.9-4.4) |  | 4.3 (4.2-4.5) |
| Kenya | 2014 | Lower-middle | 6.8 (6.6-7.0) |  | 6.8 (6.8-6.9) |  | 7.0 (6.9-7.0) |
| Angola | 2015 | Upper-middle | 4.8 (4.3-5.2) |  | 6.0 (5.8-6.2) |  | 6.3 (6.2-6.5) |
| Burundi | 2016 | Low | 5.6 (5.5-5.7) |  | 5.7 (5.7-5.8) |  | 5.9 (5.8-5.9) |
| Zambia | 2013 | Lower-middle | 6.0 (5.8-6.1) |  | 6.1 (6.0-6.1) |  | 6.3 (6.3-6.4) |
| Tanzania | 2015 | Low | 5.8 (5.6-5.9) |  | 6.2 (6.1-6.3) |  | 6.5 (6.4-6.6) |
| Uganda | 2016 | Low | 6.3 (6.2-6.4) |  | 6.4 (6.3-6.4) |  | 6.5 (6.4-6.6) |
| Malawi | 2015 | Low | 6.3 (6.2-6.4) |  | 6.4 (6.3-6.4) |  | 6.4 (6.3-6.4) |
| Rwanda | 2014 | Low | 6.7 (6.5-6.8) |  | 6.6 (6.5-6.7) |  | 6.7 (6.6-6.7) |
| Comoros | 2012 | Low | 6.3 (6.0-6.5) |  | 6.7 (6.5-7.0) |  | 7.2 (6.9-7.5) |
| Zimbabwe | 2015 | Low | 5.7 (5.1-6.4) |  | 6.5 (6.3-6.7) |  | 7.1 (7.0-7.2) |
| Lesotho | 2014 | Lower-middle | 7.3 (6.9-7.8) |  | 7.5 (7.3-7.7) |  | 7.6 (7.5-7.7) |
| South Africa | 2016 | Upper-middle | 6.7 (5.9-7.4) |  | 7.2 (6.6-7.8) |  | 7.3 (7.2-7.5) |
| Namibia | 2013 | Upper-middle | 6.3 (5.4-7.1) |  | 7.2 (6.9-7.4) |  | 7.6 (7.5-7.7) |
| **Middle East & North Africa** | |  |  |  |  |  |  |
| Egypt | 2014 | Lower-middle | 5.7 (5.6-5.9) |  | 6.2 (6.1-6.3) |  | 6.8 (6.7-6.8) |
| **South Asia** |  |  |  |  |  |  |  |
| Afghanistan | 2015 | Low | 3.0 (2.8-3.1) |  | 3.8 (3.7-4.0) |  | 3.7 (3.6-3.8) |
| Pakistan | 2017 | Lower-middle | 5.9 (5.8-6.1) |  | 6.6 (6.4-6.8) |  | 7.0 (6.8-7.1) |
| India | 2015 | Lower-middle | 6.3 (6.2-6.4) |  | 6.8 (6.7-6.9) |  | 7.1 (7.1-7.2) |
| Nepal | 2016 | Low | 6.7 (6.6-6.8) |  | 7.0 (6.8-7.2) |  | 6.9 (6.7-7.1) |
| **East Asia & the Pacific** | |  |  |  |  |  |  |
| Myanmar | 2015 | Lower-middle | 5.3 (4.8-5.8) |  | 6.1 (5.9-6.3) |  | 6.3 (6.2-6.5) |
| Timor Leste | 2016 | Lower-middle | 5.4 (4.6-6.1) |  | 6.6 (6.3-6.9) |  | 6.6 (6.5-6.7) |
| Indonesia | 2012 | Lower-middle | 6.9 (6.7-7.1) |  | 7.3 (7.2-7.4) |  | 7.4 (7.4-7.5) |
| Cambodia | 2014 | Low | 7.0 (6.6-7.4) |  | 7.4 (7.2-7.5) |  | 7.3 (7.2-7.4) |
| Philippines | 2017 | Lower-middle | 7.3 (6.9-7.6) |  | 7.4 (7.2-7.6) |  | 7.8 (7.7-7.8) |
| **Latin America & Caribbean** | |  |  |  |  |  |  |
| Haiti | 2016 | Low | 7.2 (6.9-7.5) |  | 7.1 (6.9-7.2) |  | 7.3 (7.2-7.4) |
| Guatemala | 2014 | Lower-middle | 6.5 (6.2-6.8) |  | 7.5 (7.3-7.6) |  | 7.8 (7.7-7.8) |
| Honduras | 2011 | Lower-middle | 7.4 (7.1-7.6) |  | 7.8 (7.7-7.9) |  | 8.2 (8.1-8.2) |
| Peru | 2016 | Upper-middle | 8.1 (7.8-8.3) |  | 8.7 (8.6-8.8) |  | 9.0 (8.9-9.0) |

**Table S6.** Means and 95% confidence intervals of ANCq score by place of residence. Source: DHS and MICS, 2010-2017.

| **Country** | **Year** | **Source** | **Income Group** | **Place of residence** | | |
| --- | --- | --- | --- | --- | --- | --- |
|  |  |  |  | **Urban** |  | **Rural** |
|  |  |  |  | **ANCq Mean (95%CI)** |  | **ANCq Mean (95%CI)** |
| **West & Central Africa** | |  |  |  |  |  |
| Chad | 2014 | DHS | Low | 6.0 (5.7-6.3) |  | 3.4 (3.2-3.6) |
| Congo DR | 2013 | DHS | Low | 6.4 (6.3-6.5) |  | 5.0 (4.8-5.1) |
| Niger | 2012 | DHS | Low | 6.9 (6.7-7.0) |  | 4.4 (4.3-4.6) |
| Mali | 2015 | MICS | Low | 7.2 (6.9-7.5) |  | 5.4 (5.3-5.5) |
| Burkina Faso | 2010 | DHS | Low | 7.4 (7.3-7.5) |  | 6.4 (6.3-6.5) |
| Gambia | 2013 | DHS | Low | 7.4 (7.3-7.5) |  | 7.5 (7.4-7.6) |
| Togo | 2013 | DHS | Low | 7.5 (7.4-7.7) |  | 5.7 (5.5-5.8) |
| Benin | 2014 | MICS | Low | 7.6 (7.4-7.7) |  | 7.0 (7.0-7.1) |
| Côte d'Ivoire | 2016 | MICS | Lower-middle | 7.6 (7.5-7.7) |  | 6.9 (6.8-6.9) |
| Guinea Bissau | 2014 | MICS | Low | 7.8 (7.7-7.9) |  | 7.2 (7.1-7.3) |
| Senegal | 2017 | DHS | Low | 7.8 (7.8-7.9) |  | 7.0 (6.9-7.1) |
| Mauritania | 2015 | MICS | Lower-middle | 7.9 (7.8-8.0) |  | 7.0 (6.9-7.1) |
| Nigeria | 2016 | MICS | Lower-middle | 7.9 (7.9-8.0) |  | 7.1 (7.0-7.2) |
| Cameroon | 2014 | MICS | Lower-middle | 8.0 (7.9-8.0) |  | 7.3 (7.2-7.4) |
| Gabon | 2012 | DHS | Upper-middle | 8.0 (7.9-8.1) |  | 6.7 (6.5-7.0) |
| Guinea | 2016 | MICS | Low | 8.0 (7.9-8.2) |  | 6.4 (6.2-6.5) |
| Congo | 2014 | MICS | Lower-middle | 8.1 (8.0-8.2) |  | 7.3 (7.2-7.4) |
| São Tome & Principe | 2014 | MICS | Lower-middle | 8.4 (8.3-8.6) |  | 8.3 (8.1-8.4) |
| Liberia | 2013 | DHS | Low | 8.4 (8.3-8.5) |  | 7.7 (7.5-7.9) |
| Ghana | 2014 | DHS | Lower-middle | 8.5 (8.4-8.6) |  | 7.9 (7.7-8.0) |
| **Eastern & Southern Africa** | | |  |  |  |  |
| Kenya | 2014 | DHS | Lower-middle | 5.5 (5.5-5.6) |  | 4.9 (4.9-5.0) |
| Zambia | 2013 | DHS | Lower-middle | 6.6 (6.5-6.6) |  | 5.9 (5.9-6.0) |
| Malawi | 2015 | DHS | Low | 6.8 (6.6-6.9) |  | 6.3 (6.2-6.3) |
| Burundi | 2016 | DHS | Low | 6.8 (6.7-7.0) |  | 5.7 (5.6-5.7) |
| Uganda | 2016 | DHS | Low | 6.9 (6.8-7.0) |  | 6.3 (6.2-6.3) |
| Ethiopia | 2016 | DHS | Low | 6.9 (6.5-7.3) |  | 3.7 (3.5-4.0) |
| Rwanda | 2014 | DHS | Low | 6.9 (6.8-7.0) |  | 6.6 (6.5-6.6) |
| Comoros | 2012 | DHS | Low | 7.1 (6.9-7.3) |  | 6.5 (6.3-6.8) |
| Zimbabwe | 2015 | DHS | Low | 7.1 (7.0-7.3) |  | 6.8 (6.6-6.9) |
| Tanzania | 2015 | DHS | Low | 7.2 (7.1-7.3) |  | 5.9 (5.8-6.0) |
| South Africa | 2016 | DHS | Upper-middle | 7.2 (7.0-7.4) |  | 7.4 (7.3-7.5) |
| Angola | 2015 | DHS | Upper-middle | 7.2 (7.1-7.4) |  | 4.2 (3.9-4.4) |
| Namibia | 2013 | DHS | Upper-middle | 7.6 (7.5-7.7) |  | 7.4 (7.2-7.5) |
| Lesotho | 2014 | DHS | Lower-middle | 7.8 (7.6-8.0) |  | 7.1 (7.0-7.3) |
| Eswatini | 2014 | MICS | Lower-middle | 8.0 (7.8-8.1) |  | 7.8 (7.7-7.9) |
| **Middle East & North Africa** | | |  |  |  |  |
| Yemen | 2013 | DHS | Lower-middle | 5.6 (5.4-5.8) |  | 3.3 (3.1-3.4) |
| Egypt | 2014 | DHS | Lower-middle | 7.0 (6.9-7.1) |  | 6.3 (6.2-6.4) |
| Sudan | 2014 | MICS | Lower-middle | 7.7 (7.6-7.8) |  | 6.9 (6.8-7.0) |
| Jordan | 2017 | DHS | Upper-middle | 8.3 (8.2-8.4) |  | 8.3 (8.2-8.4) |
| **South Asia** |  |  |  |  |  |  |
| Afghanistan | 2015 | DHS | Low | 4.4 (4.2-4.7) |  | 3.2 (2.9-3.4) |
| Nepal | 2016 | DHS | Low | 7.3 (7.1-7.5) |  | 6.3 (6.1-6.6) |
| Pakistan | 2017 | DHS | Lower-middle | 7.6 (7.4-7.9) |  | 5.8 (5.5-6.0) |
| India | 2015 | DHS | Lower-middle | 7.7 (7.7-7.8) |  | 6.3 (6.2-6.3) |
| Maldives | 2016 | DHS | Upper-middle | 9.1 (9.0-9.2) |  | 9.0 (9.0-9.1) |
| **East Asia & the Pacific** | |  |  |  |  |  |
| Timor Leste | 2016 | DHS | Lower-middle | 7.7 (7.4-7.9) |  | 6.0 (5.9-6.2) |
| Indonesia | 2012 | DHS | Lower-middle | 7.8 (7.7-7.8) |  | 7.0 (6.9-7.1) |
| Myanmar | 2015 | DHS | Lower-middle | 7.8 (7.6-8.1) |  | 5.6 (5.4-5.9) |
| Philippines | 2017 | DHS | Lower-middle | 8.0 (7.8-8.2) |  | 7.4 (7.3-7.6) |
| Cambodia | 2014 | DHS | Low | 8.1 (7.9-8.2) |  | 7.2 (7.0-7.3) |
| Vietnam | 2013 | MICS | Lower-middle | 8.4 (8.2-8.5) |  | 7.5 (7.3-7.7) |
| Thailand | 2015 | MICS | Upper-middle | 8.9 (8.8-9.0) |  | 8.8 (8.7-9.0) |
| **Latin America & Caribbean** | | |  |  |  |  |
| Haiti | 2016 | DHS | Low | 7.7 (7.5-7.9) |  | 6.9 (6.7-7.1) |
| Guatemala | 2014 | DHS | Lower-middle | 8.0 (7.9-8.1) |  | 7.3 (7.2-7.4) |
| Honduras | 2011 | DHS | Lower-middle | 8.2 (8.2-8.3) |  | 7.7 (7.6-7.7) |
| Guyana | 2014 | MICS | Lower-middle | 8.2 (8.1-8.4) |  | 7.9 (7.8-8.0) |
| El Salvador | 2014 | MICS | Lower-middle | 8.6 (8.6-8.7) |  | 8.4 (8.4-8.5) |
| Colombia | 2015 | DHS | Upper-middle | 8.7 (8.6-8.7) |  | 7.9 (7.8-8.1) |
| Mexico | 2015 | MICS | Upper-middle | 8.7 (8.6-8.8) |  | 8.5 (8.4-8.7) |
| Belize | 2015 | MICS | Upper-middle | 8.8 (8.6-8.9) |  | 8.5 (8.3-8.6) |
| Peru | 2016 | DHS | Upper-middle | 9.0 (8.9-9.0) |  | 8.4 (8.3-8.5) |
| Paraguay | 2016 | MICS | Upper-middle | 9.2 (9.1-9.3) |  | 8.8 (8.7-8.9) |
| Dominican Rep | 2014 | MICS | Upper-middle | 9.3 (9.2-9.3) |  | 9.2 (9.2-9.3) |
| Cuba | 2014 | MICS | Upper-middle | 9.3 (9.2-9.4) |  | 9.2 (9.1-9.4) |

**Table S7.** Means and 95% confidence intervals of ANCq score by woman’s education level. Source: DHS and MICS, 2010-2017.

| **Country** | **Year** | **Source** | **Income Group** | **Woman's educational level** | | | | |
| --- | --- | --- | --- | --- | --- | --- | --- | --- |
|  |  |  |  | **None** |  | **Primary** |  | **Secondary+** |
|  |  |  |  | **ANCq Mean (95%CI)** |  | **ANCq Mean (95%CI)** |  | **ANCq Mean (95%CI)** |
| **West & Central Africa** | |  |  |  |  |  |  |  |
| Chad | 2014 | DHS | Low | 3.1 (2.9-3.3) |  | 5.1 (4.9-5.3) |  | 6.4 (6.2-6.6) |
| Congo DR | 2013 | DHS | Low | 4.5 (4.3-4.8) |  | 5.1 (4.9-5.2) |  | 6.3 (6.2-6.3) |
| Niger | 2012 | DHS | Low | 4.6 (4.4-4.7) |  | 5.6 (5.4-5.8) |  | 6.9 (6.7-7.1) |
| Mali | 2015 | MICS | Low | 5.5 (5.4-5.6) |  | 6.2 (5.9-6.4) |  | 6.7 (6.5-6.9) |
| Togo | 2013 | DHS | Low | 5.6 (5.3-5.8) |  | 6.5 (6.4-6.7) |  | 7.4 (7.3-7.5) |
| Burkina Faso | 2010 | DHS | Low | 6.4 (6.3-6.5) |  | 7.1 (7.0-7.2) |  | 7.7 (7.6-7.8) |
| Guinea | 2016 | MICS | Low | 6.7 (6.6-6.8) |  | 7.3 (7.1-7.4) |  | 7.9 (7.7-8.1) |
| Nigeria | 2016 | MICS | Lower-middle | 6.8 (6.7-6.9) |  | 7.2 (7.1-7.3) |  | 7.8 (7.8-7.9) |
| Cameroon | 2014 | MICS | Lower-middle | 7.0 (6.8-7.2) |  | 7.5 (7.4-7.6) |  | 8.0 (8.0-8.1) |
| Benin | 2014 | MICS | Low | 7.0 (6.9-7.1) |  | 7.5 (7.3-7.6) |  | 7.8 (7.6-7.9) |
| Côte d'Ivoire | 2016 | MICS | Lower-middle | 7.0 (6.9-7.1) |  | 7.2 (7.0-7.3) |  | 7.6 (7.4-7.8) |
| Senegal | 2017 | DHS | Low | 7.0 (6.9-7.1) |  | 7.6 (7.5-7.7) |  | 8.0 (7.9-8.1) |
| Mauritania | 2015 | MICS | Lower-middle | 7.2 (7.0-7.3) |  | 7.2 (7.1-7.4) |  | 7.4 (7.3-7.5) |
| Guinea Bissau | 2014 | MICS | Low | 7.2 (7.1-7.3) |  | 7.4 (7.3-7.5) |  | 7.9 (7.8-8.1) |
| Congo | 2014 | MICS | Lower-middle | 7.4 (7.1-7.6) |  | 7.5 (7.4-7.6) |  | 7.8 (7.7-7.9) |
| Gambia | 2013 | DHS | Low | 7.4 (7.3-7.5) |  | 7.5 (7.4-7.6) |  | 7.5 (7.4-7.7) |
| Gabon | 2012 | DHS | Upper-middle | 7.4 (6.7-8.1) |  | 7.1 (6.9-7.3) |  | 8.1 (8.0-8.2) |
| Ghana | 2014 | DHS | Lower-middle | 7.5 (7.2-7.8) |  | 7.9 (7.7-8.1) |  | 8.5 (8.5-8.6) |
| Liberia | 2013 | DHS | Low | 7.7 (7.5-7.8) |  | 8.1 (8.0-8.2) |  | 8.6 (8.5-8.7) |
| São Tome & Principe | 2014 | MICS | Lower-middle | 8.1 (7.6-8.6) |  | 8.1 (8.0-8.3) |  | 8.7 (8.6-8.9) |
| **Eastern & Southern Africa** | | |  |  |  |  |  |  |
| Ethiopia | 2016 | DHS | Low | 3.3 (3.1-3.6) |  | 4.9 (4.7-5.2) |  | 7.2 (6.9-7.4) |
| Angola | 2015 | DHS | Upper-middle | 4.1 (3.8-4.3) |  | 6.1 (5.9-6.3) |  | 7.8 (7.7-7.9) |
| Kenya | 2014 | DHS | Lower-middle | 4.3 (4.1-4.4) |  | 5.0 (5.0-5.1) |  | 5.6 (5.5-5.7) |
| Burundi | 2016 | DHS | Low | 5.5 (5.5-5.6) |  | 5.8 (5.8-5.9) |  | 6.6 (6.5-6.7) |
| Zambia | 2013 | DHS | Lower-middle | 5.6 (5.5-5.8) |  | 6.1 (6.0-6.1) |  | 6.5 (6.4-6.6) |
| Tanzania | 2015 | DHS | Low | 5.7 (5.5-5.8) |  | 6.3 (6.2-6.4) |  | 7.2 (7.0-7.3) |
| Malawi | 2015 | DHS | Low | 6.1 (6.0-6.2) |  | 6.3 (6.2-6.3) |  | 6.7 (6.6-6.8) |
| Comoros | 2012 | DHS | Low | 6.1 (5.8-6.4) |  | 6.8 (6.4-7.1) |  | 7.5 (7.3-7.7) |
| Uganda | 2016 | DHS | Low | 6.2 (6.0-6.3) |  | 6.2 (6.1-6.3) |  | 6.9 (6.8-6.9) |
| Rwanda | 2014 | DHS | Low | 6.3 (6.1-6.4) |  | 6.6 (6.5-6.7) |  | 7.2 (7.1-7.3) |
| Namibia | 2013 | DHS | Upper-middle | 6.4 (5.9-6.8) |  | 7.1 (6.9-7.3) |  | 7.7 (7.6-7.8) |
| Lesotho | 2014 | DHS | Lower-middle | 6.5 (5.3-7.7) |  | 7.0 (6.8-7.2) |  | 7.6 (7.5-7.7) |
| Zimbabwe | 2015 | DHS | Low | 6.5 (5.9-7.1) |  | 6.5 (6.2-6.8) |  | 7.1 (7.0-7.2) |
| South Africa | 2016 | DHS | Upper-middle | 7.5 (7.0-8.1) |  | 7.1 (6.6-7.5) |  | 7.3 (7.2-7.4) |
| Eswatini | 2014 | MICS | Lower-middle | 8.0 (7.7-8.3) |  | 7.7 (7.5-7.8) |  | 7.9 (7.8-7.9) |
| **Middle East & North Africa** | | |  |  |  |  |  |  |
| Yemen | 2013 | DHS | Lower-middle | 3.0 (2.9-3.2) |  | 4.6 (4.4-4.7) |  | 6.0 (5.8-6.2) |
| Egypt | 2014 | DHS | Lower-middle | 5.4 (5.2-5.6) |  | 6.0 (5.7-6.2) |  | 6.8 (6.7-6.9) |
| Sudan | 2014 | MICS | Lower-middle | 6.5 (6.3-6.6) |  | 7.2 (7.1-7.3) |  | 7.9 (7.8-8.0) |
| Jordan | 2017 | DHS | Upper-middle | 7.3 (6.6-8.0) |  | 7.6 (7.3-8.0) |  | 8.4 (8.3-8.4) |
| **South Asia** |  |  |  |  |  |  |  |  |
| Afghanistan | 2015 | DHS | Low | 3.2 (3.0-3.4) |  | 4.4 (4.0-4.7) |  | 5.2 (4.9-5.5) |
| India | 2015 | DHS | Lower-middle | 4.9 (4.8-4.9) |  | 6.4 (6.3-6.4) |  | 7.6 (7.6-7.7) |
| Pakistan | 2017 | DHS | Lower-middle | 5.0 (4.8-5.2) |  | 6.8 (6.6-7.0) |  | 8.1 (8.0-8.2) |
| Nepal | 2016 | DHS | Low | 5.6 (5.4-5.9) |  | 6.5 (6.3-6.7) |  | 7.8 (7.7-7.9) |
| Maldives | 2016 | DHS | Upper-middle | 8.9 (8.1-9.6) |  | 9.0 (8.8-9.1) |  | 9.1 (9.0-9.1) |
| **East Asia & the Pacific** | |  |  |  |  |  |  |  |
| Myanmar | 2015 | DHS | Lower-middle | 4.0 (3.4-4.6) |  | 5.9 (5.6-6.1) |  | 7.4 (7.2-7.6) |
| Indonesia | 2012 | DHS | Lower-middle | 4.3 (3.7-4.9) |  | 6.9 (6.8-7.0) |  | 7.7 (7.6-7.7) |
| Philippines | 2017 | DHS | Lower-middle | 4.6 (3.8-5.4) |  | 6.4 (6.1-6.8) |  | 8.0 (7.9-8.1) |
| Timor Leste | 2016 | DHS | Lower-middle | 5.3 (5.0-5.7) |  | 6.2 (5.9-6.4) |  | 7.1 (7.0-7.3) |
| Cambodia | 2014 | DHS | Low | 6.1 (5.7-6.4) |  | 7.2 (7.1-7.3) |  | 7.9 (7.8-8.0) |
| Vietnam | 2013 | MICS | Lower-middle | 6.2 (5.5-6.9) |  | 6.9 (6.6-7.2) |  | 7.9 (7.8-8.1) |
| Thailand | 2015 | MICS | Upper-middle | 8.6 (7.8-9.4) |  | 8.7 (8.5-8.9) |  | 8.8 (8.7-8.9) |
| **Latin America & Caribbean** | | |  |  |  |  |  |  |
| Haiti | 2016 | DHS | Low | 5.7 (5.4-6.1) |  | 7.0 (6.8-7.2) |  | 8.0 (7.9-8.1) |
| Colombia | 2015 | DHS | Upper-middle | 5.7 (4.9-6.6) |  | 7.9 (7.7-8.0) |  | 8.6 (8.6-8.7) |
| Guatemala | 2014 | DHS | Lower-middle | 6.6 (6.4-6.8) |  | 7.3 (7.2-7.4) |  | 8.4 (8.3-8.5) |
| Honduras | 2011 | DHS | Lower-middle | 6.7 (6.3-7.1) |  | 7.7 (7.6-7.8) |  | 8.4 (8.4-8.5) |
| Paraguay | 2016 | MICS | Upper-middle | 7.6 (7.1-8.0) |  | 8.6 (8.5-8.8) |  | 9.0 (8.9-9.1) |
| Mexico | 2015 | MICS | Upper-middle | 7.6 (7.1-8.1) |  | 8.3 (8.1-8.5) |  | 8.7 (8.6-8.8) |
| Belize | 2015 | MICS | Upper-middle | 7.7 (7.2-8.2) |  | 8.5 (8.4-8.6) |  | 8.7 (8.5-8.8) |
| Guyana | 2014 | MICS | Lower-middle | 7.9 (7.4-8.3) |  | 7.6 (7.4-7.8) |  | 8.1 (8.0-8.1) |
| Peru | 2016 | DHS | Upper-middle | 8.0 (7.6-8.4) |  | 8.4 (8.3-8.5) |  | 9.0 (8.9-9.0) |
| El Salvador | 2014 | MICS | Lower-middle | 8.3 (8.0-8.5) |  | 8.3 (8.2-8.4) |  | 8.7 (8.6-8.8) |
| Dominican Rep | 2014 | MICS | Upper-middle | 8.7 (8.5-8.9) |  | 9.0 (9.0-9.1) |  | 9.4 (9.3-9.4) |
| Cuba | 2014 | MICS | Upper-middle |  |  | 9.2 (9.0-9.5) |  | 9.3 (9.2-9.4) |

**Table S8.** Means and 95% confidence intervals of ANCq score by woman’s age at childbirth. Source: DHS and MICS, 2010-2017.

| **Country** | **Year** | **Source** | **Income Group** | **Woman's age at childbirth** | | | | |
| --- | --- | --- | --- | --- | --- | --- | --- | --- |
|  |  |  |  | **15-19 years** |  | **20-34 years** |  | **35-49 years** |
|  |  |  |  | **ANCq Mean (95%CI)** |  | **ANCq Mean (95%CI)** |  | **ANCq Mean (95%CI)** |
| **West & Central Africa** | |  |  |  |  |  |  |  |
| Chad | 2014 | DHS | Low | 4.1 (3.9-4.3) |  | 4.0 (3.8-4.1) |  | 3.6 (3.4-3.9) |
| Niger | 2012 | DHS | Low | 4.8 (4.6-5.0) |  | 4.8 (4.7-4.9) |  | 4.7 (4.5-4.9) |
| Congo DR | 2013 | DHS | Low | 5.6 (5.4-5.7) |  | 5.5 (5.3-5.6) |  | 5.2 (5.0-5.4) |
| Mali | 2015 | MICS | Low | 5.7 (5.5-5.9) |  | 5.9 (5.7-6.0) |  | 5.8 (5.6-6.0) |
| Togo | 2013 | DHS | Low | 6.2 (5.9-6.5) |  | 6.5 (6.3-6.6) |  | 6.0 (5.7-6.2) |
| Burkina Faso | 2010 | DHS | Low | 6.7 (6.6-6.8) |  | 6.7 (6.6-6.7) |  | 6.3 (6.2-6.4) |
| Guinea | 2016 | MICS | Low | 6.9 (6.8-7.0) |  | 7.1 (7.0-7.3) |  | 7.7 (6.9-8.4) |
| Côte d'Ivoire | 2016 | MICS | Lower-middle | 7.0 (6.9-7.2) |  | 7.2 (7.1-7.3) |  | 7.0 (6.8-7.2) |
| Nigeria | 2016 | MICS | Lower-middle | 7.1 (6.9-7.2) |  | 7.5 (7.4-7.5) |  | 7.4 (7.3-7.5) |
| Benin | 2014 | MICS | Low | 7.2 (7.0-7.4) |  | 7.3 (7.2-7.4) |  | 7.1 (7.0-7.3) |
| Mauritania | 2015 | MICS | Lower-middle | 7.2 (7.1-7.4) |  | 7.5 (7.4-7.6) |  | 7.4 (7.2-7.5) |
| Guinea Bissau | 2014 | MICS | Low | 7.4 (7.2-7.5) |  | 7.4 (7.3-7.5) |  | 7.3 (7.2-7.5) |
| Cameroon | 2014 | MICS | Lower-middle | 7.4 (7.3-7.6) |  | 7.7 (7.6-7.8) |  | 7.4 (7.3-7.6) |
| Senegal | 2017 | DHS | Low | 7.4 (7.3-7.5) |  | 7.4 (7.3-7.5) |  | 7.1 (7.0-7.2) |
| Gambia | 2013 | DHS | Low | 7.5 (7.4-7.6) |  | 7.4 (7.4-7.5) |  | 7.4 (7.3-7.5) |
| Gabon | 2012 | DHS | Upper-middle | 7.7 (7.5-7.9) |  | 7.9 (7.8-8.0) |  | 7.6 (7.3-7.8) |
| Congo | 2014 | MICS | Lower-middle | 7.7 (7.7-7.8) |  | 8.0 (7.9-8.0) |  | 7.7 (6.9-8.5) |
| Ghana | 2014 | DHS | Lower-middle | 8.0 (7.7-8.2) |  | 8.2 (8.1-8.3) |  | 8.0 (7.9-8.2) |
| Liberia | 2013 | DHS | Low | 8.1 (7.9-8.2) |  | 8.1 (8.0-8.2) |  | 8.1 (7.9-8.3) |
| São Tome & Principe | 2014 | MICS | Lower-middle | 8.4 (8.2-8.7) |  | 8.4 (8.3-8.5) |  | 8.0 (7.8-8.3) |
| **Eastern & Southern Africa** | | |  |  |  |  |  |  |
| Ethiopia | 2016 | DHS | Low | 4.3 (3.9-4.6) |  | 4.3 (4.0-4.5) |  | 3.4 (3.1-3.8) |
| Kenya | 2014 | DHS | Lower-middle | 5.0 (4.8-5.1) |  | 5.2 (5.2-5.3) |  | 5.0 (4.8-5.1) |
| Angola | 2015 | DHS | Upper-middle | 5.9 (5.7-6.1) |  | 6.3 (6.2-6.5) |  | 5.7 (5.4-6.0) |
| Comoros | 2012 | DHS | Low | 6.1 (5.7-6.5) |  | 6.9 (6.7-7.1) |  | 6.5 (6.1-6.8) |
| Burundi | 2016 | DHS | Low | 6.1 (6.0-6.2) |  | 5.8 (5.8-5.9) |  | 5.5 (5.4-5.6) |
| Zambia | 2013 | DHS | Lower-middle | 6.1 (6.0-6.2) |  | 6.2 (6.2-6.3) |  | 6.0 (5.9-6.1) |
| Tanzania | 2015 | DHS | Low | 6.3 (6.1-6.4) |  | 6.4 (6.3-6.5) |  | 6.0 (5.8-6.1) |
| Malawi | 2015 | DHS | Low | 6.3 (6.2-6.4) |  | 6.4 (6.3-6.4) |  | 6.3 (6.2-6.4) |
| Uganda | 2016 | DHS | Low | 6.3 (6.2-6.4) |  | 6.5 (6.4-6.6) |  | 6.1 (6.0-6.2) |
| Zimbabwe | 2015 | DHS | Low | 6.8 (6.6-7.0) |  | 6.9 (6.7-7.0) |  | 7.0 (6.8-7.3) |
| Rwanda | 2014 | DHS | Low | 6.9 (6.8-7.0) |  | 6.7 (6.6-6.7) |  | 6.3 (6.2-6.4) |
| Namibia | 2013 | DHS | Upper-middle | 7.1 (6.9-7.3) |  | 7.6 (7.5-7.7) |  | 7.5 (7.3-7.7) |
| South Africa | 2016 | DHS | Upper-middle | 7.2 (6.9-7.4) |  | 7.3 (7.2-7.5) |  | 7.2 (6.9-7.4) |
| Lesotho | 2014 | DHS | Lower-middle | 7.3 (7.1-7.4) |  | 7.4 (7.3-7.5) |  | 7.0 (6.7-7.3) |
| Eswatini | 2014 | MICS | Lower-middle | 7.6 (7.4-7.8) |  | 7.9 (7.8-8.0) |  | 7.9 (7.7-8.1) |
| **Middle East & North Africa** | | |  |  |  |  |  |  |
| Yemen | 2013 | DHS | Lower-middle | 4.2 (4.0-4.5) |  | 4.0 (3.9-4.2) |  | 3.4 (3.2-3.6) |
| Egypt | 2014 | DHS | Lower-middle | 6.3 (6.0-6.5) |  | 6.5 (6.4-6.6) |  | 6.4 (6.2-6.6) |
| Sudan | 2014 | MICS | Lower-middle | 7.2 (7.0-7.4) |  | 7.2 (7.1-7.2) |  | 7.1 (6.9-7.2) |
| Jordan | 2017 | DHS | Upper-middle | 8.3 (8.1-8.5) |  | 8.3 (8.2-8.4) |  | 8.3 (8.2-8.4) |
| **South Asia** |  |  |  |  |  |  |  |  |
| Afghanistan | 2015 | DHS | Low | 3.5 (3.2-3.8) |  | 3.4 (3.2-3.6) |  | 3.5 (3.2-3.9) |
| Pakistan | 2017 | DHS | Lower-middle | 6.0 (5.7-6.4) |  | 6.6 (6.4-6.8) |  | 5.7 (5.3-6.0) |
| India | 2015 | DHS | Lower-middle | 6.9 (6.9-7.0) |  | 6.8 (6.7-6.8) |  | 5.1 (5.0-5.2) |
| Nepal | 2016 | DHS | Low | 7.2 (7.0-7.4) |  | 6.9 (6.7-7.0) |  | 5.2 (4.5-5.8) |
| Maldives | 2016 | DHS | Upper-middle | 9.2 (8.9-9.4) |  | 9.1 (9.0-9.1) |  | 9.0 (8.7-9.2) |
| **East Asia & the Pacific** | |  |  |  |  |  |  |  |
| Myanmar | 2015 | DHS | Lower-middle | 5.5 (5.0-6.0) |  | 6.3 (6.1-6.5) |  | 5.9 (5.6-6.3) |
| Timor Leste | 2016 | DHS | Lower-middle | 6.2 (5.8-6.6) |  | 6.7 (6.5-6.8) |  | 6.1 (5.8-6.3) |
| Indonesia | 2012 | DHS | Lower-middle | 7.1 (6.9-7.3) |  | 7.4 (7.4-7.5) |  | 7.1 (7.0-7.3) |
| Cambodia | 2014 | DHS | Low | 7.2 (7.0-7.4) |  | 7.4 (7.3-7.5) |  | 6.4 (6.1-6.7) |
| Vietnam | 2013 | MICS | Lower-middle | 7.3 (6.9-7.7) |  | 7.8 (7.7-7.9) |  | 7.7 (7.4-8.1) |
| Philippines | 2017 | DHS | Lower-middle | 7.4 (7.2-7.7) |  | 7.8 (7.7-7.9) |  | 7.6 (7.4-7.8) |
| Thailand | 2015 | MICS | Upper-middle | 8.5 (8.3-8.7) |  | 9.0 (8.9-9.1) |  | 8.9 (8.7-9.1) |
| **Latin America & Caribbean** | | |  |  |  |  |  |  |
| Haiti | 2016 | DHS | Low | 6.9 (6.7-7.2) |  | 7.4 (7.2-7.5) |  | 6.8 (6.5-7.0) |
| Guatemala | 2014 | DHS | Lower-middle | 7.5 (7.3-7.7) |  | 7.6 (7.5-7.7) |  | 7.1 (7.0-7.3) |
| Honduras | 2011 | DHS | Lower-middle | 7.8 (7.7-7.9) |  | 8.0 (8.0-8.1) |  | 7.6 (7.5-7.8) |
| Guyana | 2014 | MICS | Lower-middle | 7.9 (7.8-8.1) |  | 8.0 (7.9-8.1) |  | 7.8 (7.6-8.1) |
| Colombia | 2015 | DHS | Upper-middle | 8.1 (8.0-8.2) |  | 8.5 (8.5-8.6) |  | 8.6 (8.4-8.8) |
| El Salvador | 2014 | MICS | Lower-middle | 8.4 (8.3-8.5) |  | 8.6 (8.6-8.7) |  | 8.5 (8.3-8.7) |
| Mexico | 2015 | MICS | Upper-middle | 8.5 (8.3-8.6) |  | 8.7 (8.6-8.8) |  | 8.5 (8.1-8.9) |
| Peru | 2016 | DHS | Upper-middle | 8.5 (8.4-8.6) |  | 8.9 (8.9-8.9) |  | 8.8 (8.8-8.9) |
| Belize | 2015 | MICS | Upper-middle | 8.6 (8.3-8.8) |  | 8.6 (8.5-8.8) |  | 8.4 (8.2-8.6) |
| Paraguay | 2016 | MICS | Upper-middle | 8.7 (8.5-8.9) |  | 9.1 (9.0-9.2) |  | 9.0 (8.8-9.3) |
| Dominican Rep | 2014 | MICS | Upper-middle | 9.1 (9.1-9.2) |  | 9.3 (9.3-9.3) |  | 9.3 (9.2-9.4) |
| Cuba | 2014 | MICS | Upper-middle | 9.2 (9.0-9.4) |  | 9.3 (9.2-9.4) |  | 9.3 (9.2-9.5) |

**Table S9.** Means and 95% confidence intervals of ANCq score by sex of the child. Source: DHS and MICS, 2010-2017.

| **Country** | **Year** | **Source** | **Income Group** | **Sex of the child** | | |
| --- | --- | --- | --- | --- | --- | --- |
|  |  |  |  | **Female** |  | **Male** |
|  |  |  |  | **ANCq Mean (95%CI)** |  | **ANCq Mean (95%CI)** |
| **West & Central Africa** | |  |  |  |  |  |
| Chad | 2014 | DHS | Low | 3.9 (3.7-4.0) |  | 4.0 (3.8-4.2) |
| Niger | 2012 | DHS | Low | 4.9 (4.7-5.0) |  | 4.7 (4.6-4.9) |
| Congo DR | 2013 | DHS | Low | 5.5 (5.4-5.6) |  | 5.4 (5.3-5.5) |
| Togo | 2013 | DHS | Low | 6.3 (6.2-6.5) |  | 6.4 (6.2-6.5) |
| Burkina Faso | 2010 | DHS | Low | 6.6 (6.5-6.7) |  | 6.6 (6.5-6.7) |
| Senegal | 2017 | DHS | Low | 7.4 (7.3-7.4) |  | 7.3 (7.2-7.4) |
| Gambia | 2013 | DHS | Low | 7.4 (7.3-7.5) |  | 7.5 (7.4-7.6) |
| Gabon | 2012 | DHS | Upper-middle | 7.8 (7.7-7.9) |  | 7.9 (7.7-8.0) |
| Liberia | 2013 | DHS | Low | 8.1 (8.0-8.2) |  | 8.1 (8.0-8.2) |
| Ghana | 2014 | DHS | Lower-middle | 8.2 (8.1-8.3) |  | 8.1 (8.0-8.3) |
| **Eastern & Southern Africa** | | |  |  |  |  |
| Ethiopia | 2016 | DHS | Low | 4.1 (3.9-4.4) |  | 4.1 (3.9-4.3) |
| Kenya | 2014 | DHS | Lower-middle | 5.2 (5.1-5.2) |  | 5.2 (5.1-5.2) |
| Burundi | 2016 | DHS | Low | 5.8 (5.7-5.8) |  | 5.8 (5.7-5.8) |
| Angola | 2015 | DHS | Upper-middle | 6.1 (6.0-6.3) |  | 6.1 (6.0-6.3) |
| Zambia | 2013 | DHS | Lower-middle | 6.2 (6.1-6.3) |  | 6.2 (6.1-6.2) |
| Tanzania | 2015 | DHS | Low | 6.3 (6.2-6.4) |  | 6.3 (6.2-6.4) |
| Malawi | 2015 | DHS | Low | 6.3 (6.3-6.4) |  | 6.4 (6.3-6.5) |
| Uganda | 2016 | DHS | Low | 6.4 (6.4-6.5) |  | 6.4 (6.3-6.4) |
| Rwanda | 2014 | DHS | Low | 6.6 (6.6-6.7) |  | 6.6 (6.6-6.7) |
| Comoros | 2012 | DHS | Low | 6.8 (6.6-7.0) |  | 6.6 (6.4-6.9) |
| Zimbabwe | 2015 | DHS | Low | 6.9 (6.8-7.0) |  | 6.8 (6.7-7.0) |
| South Africa | 2016 | DHS | Upper-middle | 7.2 (7.1-7.4) |  | 7.3 (7.2-7.5) |
| Lesotho | 2014 | DHS | Lower-middle | 7.4 (7.3-7.5) |  | 7.2 (7.1-7.4) |
| Namibia | 2013 | DHS | Upper-middle | 7.5 (7.4-7.6) |  | 7.5 (7.3-7.6) |
| **Middle East & North Africa** | | |  |  |  |  |
| Yemen | 2013 | DHS | Lower-middle | 4.0 (3.8-4.1) |  | 3.9 (3.8-4.1) |
| Egypt | 2014 | DHS | Lower-middle | 6.4 (6.3-6.5) |  | 6.6 (6.5-6.7) |
| Jordan | 2017 | DHS | Upper-middle | 8.3 (8.2-8.4) |  | 8.3 (8.2-8.4) |
| **South Asia** |  |  |  |  |  |  |
| Afghanistan | 2015 | DHS | Low | 3.4 (3.2-3.6) |  | 3.5 (3.3-3.7) |
| Pakistan | 2017 | DHS | Lower-middle | 6.5 (6.3-6.7) |  | 6.4 (6.1-6.6) |
| India | 2015 | DHS | Lower-middle | 6.7 (6.7-6.7) |  | 6.7 (6.7-6.7) |
| Nepal | 2016 | DHS | Low | 6.8 (6.6-7.0) |  | 6.9 (6.8-7.1) |
| Maldives | 2016 | DHS | Upper-middle | 9.0 (9.0-9.1) |  | 9.0 (9.0-9.1) |
| **East Asia & the Pacific** | |  |  |  |  |  |
| Myanmar | 2015 | DHS | Lower-middle | 6.1 (5.8-6.3) |  | 6.2 (6.0-6.5) |
| Timor Leste | 2016 | DHS | Lower-middle | 6.5 (6.3-6.7) |  | 6.6 (6.4-6.8) |
| Cambodia | 2014 | DHS | Low | 7.2 (7.1-7.4) |  | 7.4 (7.2-7.5) |
| Indonesia | 2012 | DHS | Lower-middle | 7.4 (7.3-7.4) |  | 7.4 (7.3-7.4) |
| Philippines | 2017 | DHS | Lower-middle | 7.7 (7.6-7.8) |  | 7.7 (7.6-7.9) |
| **Latin America & Caribbean** | | |  |  |  |  |
| Haiti | 2016 | DHS | Low | 7.2 (7.0-7.3) |  | 7.2 (7.1-7.4) |
| Guatemala | 2014 | DHS | Lower-middle | 7.5 (7.4-7.6) |  | 7.5 (7.4-7.6) |
| Honduras | 2011 | DHS | Lower-middle | 7.9 (7.8-8.0) |  | 7.9 (7.9-8.0) |
| Colombia | 2015 | DHS | Upper-middle | 8.5 (8.4-8.6) |  | 8.5 (8.4-8.5) |
| Peru | 2016 | DHS | Upper-middle | 8.9 (8.8-8.9) |  | 8.8 (8.8-8.9) |
